# Prevention of Enzymatic Browning by Natural Extracts and Genome-Editing: A Review on Recent Progress

**DOI:** 10.3390/molecules27031101

**Published:** 2022-02-07

**Authors:** Norfadilah Hamdan, Chia Hau Lee, Syie Luing Wong, Che Ellysa Nurshafika Che Ahmad Fauzi, Nur Mirza Aqilah Zamri, Ting Hun Lee

**Affiliations:** 1 School of Chemical and Energy Engineering, Faculty of Engineering, Universiti Teknologi Malaysia, Skudai 81310, Johor, Malaysia; fadilahamdan87@gmail.com (N.H.); leechiahau0506@gmail.com (C.H.L.); syieluing@hotmail.com (S.L.W.); ellysafauzi@gmail.com (C.E.N.C.A.F.); mirzaqilah98@gmail.com (N.M.A.Z.); 2 Department of Matem’atica Aplicada, Ciencia e Ingeniería de Materiales y Tecnología Electronica, Universidad Rey Juan Carlos, C/Tulip’an s/n, M´ostoles, 28933 Madrid, Spain; 3 Innovation Centre in Agritechnology for Advanced Bioprocessing (ICA), Universiti Teknologi Malaysia, Pagoh 84600, Johor, Malaysia

**Keywords:** natural extracts, genome-editing, enzymatic browning, anti-browning

## Abstract

Fresh fruits and vegetable products are easily perishable during postharvest handling due to enzymatic browning reactions. This phenomenon has contributed to a significant loss of food quality and appearance. Thus, a safe and effective alternative method from natural sources is needed to tackle enzymatic browning prevention. The capabilities of natural anti-browning agents derived from plant- and animal-based resources in inhibiting enzymatic activity have been demonstrated in the literature. Some also possess strong antioxidants properties. This review aims to summarize a recent investigation regarding the use of natural anti-browning extracts from different sources for controlling the browning. The potential applications of genome-editing in preventing browning activity and improving postharvest quality is also discussed. Moreover, the patents on the anti-browning extract from natural sources is also presented in this review. The information reviewed here could provide new insights, contributing to the development of natural anti-browning extracts and genome-editing techniques for the prevention of food browning.

## 1. Introduction

The browning process is a natural phenomenon occurring in fruit and vegetable, which has become a challenge in the food industry sector. The undesired condition caused by browning processes has a significant impact on food quality as it causes deterioration in nutritional and sensory properties, as well as safety [1]. The food product appearance is one of the main concerns of consumers. Therefore, the colour appearance of the food product is one of the indicators considered by consumers in their selection process. The occurrence of food browning reactions usually impairs the colour appearance of food and markedly reduced the customer’s acceptance of the products [2]. Scientists and food technologists have voiced their concerns regarding the long-term impacts brought by the browning effect on fruit and vegetables, which could affect the economic situation of a country [3].

The pigmentation process that occurs in fruit and vegetable can be either enzymatic or non-enzymatic browning, depending on the mechanism [4]. Non-enzymatic browning is the term for a process that produces brown pigmentation in foods without the involvement of any enzyme activity and is associated with chemical reactions such as the Maillard reaction, caramelization, and the ascorbic acid browning reaction [5]. Natural browning of fresh fruit or vegetable occurs via an enzymatic browning mechanism. A major concern is enzymatic browning, as requires the action of enzymes present in food and oxidation in order to occur [1]. The enzymatic browning of fruit and vegetables is not desirable and causes heavy economic losses to both food producers and the food-processing industry as this process mostly occurs during postharvest handling, transportation, storage and processing [3]. Consequently, enzymatic browning affects not merely the colour alternation but also leads to nutritional and organoleptic losses, leading to less marketability and a lower consumer acceptance. Consequently, enzymatic browning development may be responsible for more than 50% of foods susceptible to browning are wasted [6].

An enzymatic browning reaction is mainly caused by enzyme polyphenol oxidase (PPO) (EC 1.10.3.1) or other enzymes such as peroxidase (POD) (EC 1.11.1.7) and tyrosinase (EC 1.14.18.1) [7,8]. These enzymes are responsible for the browning, and they can be found in nature, including in fungi, bacteria and higher plants (mushroom, apple, potato, pear, banana, peach and avocado) [1]. In the presence of oxygen, enzymatic browning takes place when these enzymes catalyze the oxidation of phenols to quinones. Subsequently, these quinones then react with other compounds and polymerize into brown pigments known as melanin [2]. Melanin pigment (dark brown) is presented in the colour of hair, irises and skin of humans. It also turns fruit and vegetables brown. This reaction, however, does not occur in fresh fruit and vegetable as their enzymes become separated from its substrate and phenolic compounds by cell compartments [7]. The enzymatic browning reaction is only triggered when those enzymes, phenolic compounds and oxygen come in contact with one another. This could explain the mechanical injuries and tissue damage that decrease the plant cell’s membrane integrity and thereby cause the improvement of the brown colour produced [4].

Enzymatic browning is an oxidation reaction. This reaction can be prevented by the removal of oxygen from the cut surface of fruit and vegetables [9]. Nonetheless, the browning can be recovered when oxygen is re-established. Therefore, the utilization of antioxidants and browning inhibitors to inactivate the enzymes responsible for browning are widely employed in the food industry, especially the fresh-cut industry [2,10]. For controlling the browning effect in fresh-cut food products, antioxidant solutions such as ascorbic acid and its derivatives have been traditionally employed due to their reducing properties [11]. Still, it is less effective and often temporary. In this regard, various forms of sulfite-compounds (sulfur dioxide and sulfites) were introduced as universal inhibitors and synthetic antioxidant for controlling the enzymatic and non-enzymatic browning of food products [3]. However, the major concern is that sulfite-compounds may cause various side effects, such as allergies complications [12]. Owing to its potential health risks to sensitive individuals, the usage of sulfite-compounds as an anti-browning agent or preservative on raw fruit and vegetable was prohibited by the Food and Drug Administration (FDA) in 1986 [11]. Considering this, the search for new food additives with anti-browning properties from the natural sources that are free from any harmful side effects are greatly needed for the food-processing industry.

Recent research has reported on the natural-based anti-browning extracts in mangrove trees [13], green tea [14], roselle [15], thyme [12] and pineapple [16]. Such findings have encouraged and motivated researchers to discover potent natural extracts to replace synthetic food additives to maintain quality and extend the shelf life of fruit and vegetables. The screening of natural sources is highly recommended as they are non-toxic and have no known adverse side effects [2]. Natural sources are frequently combined with functional botanical ingredients, which can be defined as a substance that is extracted from plant parts such as flowers, fruits, leaves, seeds and roots. Natural sources have numerous useful ingredients that have the ability to control browning development, avoiding economic losses and providing high quality foods [2,9]. Therefore, the motivation if this review is to explore the possible natural extracts reported to have anti-browning properties in natural sources and highlights their potential as food preservatives.

**Figure 1:**
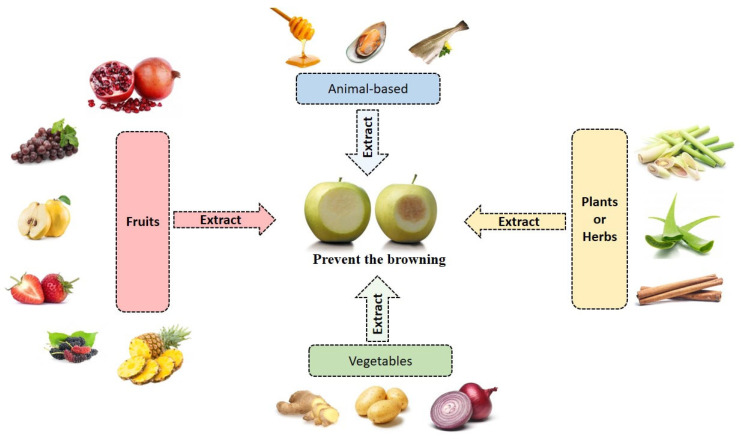
Natural extracts from fruits, vegetables, plants or herbs, and animal-based resources for preventing browning development.

Most of the current reviews focused on the technology in controlling the browning process of fruit and vegetable products [1,3,8,10]. Despite the importance of the topic, no recent review is available to provide detailed information on the application of natural-based anti-browning extract from multiple natural sources to inhibit browning in fruit and vegetable products. Hence, this current review aims to survey and summarize newly discovered enzyme inhibitors from natural sources commonly available in the market. Many of them are from the tropical region and are also widely consumed in Asian regions (including Malaysia). As shown in Figure 1, there are multiple natural extracts found in fruits, vegetables, plants/herbs, and animal by-products that are alternative resources that can be used to address the browning issues. Their natural supply, availability, and affordability make them a great tool against browning development. Meanwhile, this review also intends to discuss the potential application of genome editing in preventing browning activity and improving postharvest quality. Additionally, the patent on the anti-browning extract from natural sources is also discussed where possible. It is hoped that the knowledge offered in this review serves as an updated database contributing to the development of promising anti-browning agents from natural-source extracts. Additionally, scholars can identify the natural sources that can be used as an ingredient in browning prevention based on the outcome of this study.

### 1.1. Key Motivations of Natural-Based Anti-Browning Research

The development of brown pigment on food products (fruit and vegetable) will decrease consumer tolerability. From the consumer’s point of view, they judge the food products in terms of food’s appearance and the food’s ability to provide the taste and nutritional needs able to improve their health [8]. The increasing health care awareness has garnered government, food scientist, and the food industry attention to search for the solution and ensure the food browning problem can be handled efficiently. Searching for bioactive compounds from natural sources has become particularly attractive. The development of anti-browning agents from natural sources has been propelled by the readily available and inexpensive raw materials [2]. Bioactive compounds are functional ingredients that are found in nature or are created through the processing of foods and plants, which can provide additional beneficial effects to food products [17]. These compounds can be acquired from natural sources through physical, chemical or enzymatic processes [18]. They contain a variety of compounds from different classes with reported activities against food browning [3].

During harvesting or storage, it is very challenging to maintain the fruit and vegetables’ nutrient composition due to physiological or mechanical injury [19]. The physicochemical changes in fruit and vegetables lead to browning reactions, which reduce the visual quality and lead to the loss of conceivable valuable components [8]. When fruit and vegetables are harvested from plantation crops, their metabolic activities (rate of respiration and ethylene production) are triggered, resulting in quality deterioration [20]. According to the Food and Agriculture Organization (FAO), handling fruit and vegetables during postharvest treatments causes a 20–40% loss of fruit crops every year [21]. For example, fruits such as grapes [22], apples [23], pears [24], bananas [25], etc., and vegetables such as mushrooms [26], lettuce [27], potatoes [11], etc., are easily susceptible to browning and lead to economic losses in the food industry. To attenuate this issue, several procedures and technologies have been developed.

Currently, the existing anti-browning systems can be divided into physical and chemical approaches. Thermal treatment, modified atmosphere storage, the prevention of oxygen exposure, low temperature, and irradiation are commonly used physical-based preservation systems to prevent enzymatic browning in fruit and vegetable [1]. Still, these methods require a lot of equipment and considerable investment. Another way to suppress the enzymatic browning in fruit and vegetables is by using chemical-based methods such as antioxidant and chelating agents, but this method has potential health effects due to harmful chemical residues in the food products [3]. Most of the methods mentioned above have led to negative consequences. Therefore, the most attractive way to prevent food browning would be by natural methods. Seeking “natural” agents as food preservatives derived from plant extracts, essential oils, bioactive compounds, and purified secondary metabolites, has attracted food researchers’ attention [28,29].

**Figure 2:**
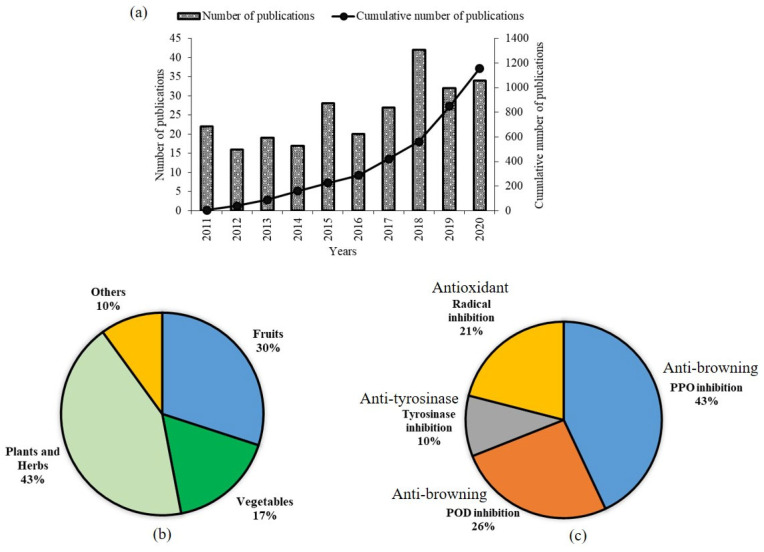
Annual and cumulative numbers of publications on the application of natural extracts in controlling enzymatic browning (**a**). Distribution of natural sources that possesses the ability to prevent enzymatic browning (**b**). Distribution of natural-based extracts with anti-browning, antioxidant and anti-tyrosinase properties (**c**). The results are based on the recent studies that determined the enzyme (PPO, POD, and tyrosinase) and radical inhibition activities of extracts derived from selected fruits, vegetables, plants/herbs and others (animal by-products), which were retrieved from WoS database.

In this review, we searched keywords such as “fruits”, “vegetables”, “plants”, “herbs”, “marine”, “animal” or “natural extracts”, combined with “anti-browning” in the Web of Science (WoS) database. Based on the search results obtained from the database, there has been a remarkable increase in the number of publications and the cumulative number of publications from 2011 to 2020 (Figure 2a). This indicates that the consumer demand for more natural and sustainable agents as food ingredients, over synthetic agents, has increased in recent years. Apart from that, natural sources of fruits (30%), vegetables (17%), plants and herbs (43%), and others (10%) are reported (Figure 2b). In this review, the mechanisms of the natural extracts in inhibiting enzymatic browning are classified into four categories, such as PPO inhibition (43%), POD inhibition (26%), tyrosinase inhibition (10%), and radical inhibition (21%) (Figure 2c). Some natural sources are reported to have multiple inhibition activities.

### 1.2. Market Potential

The demand in the natural preservatives market with active ingredients is expanding, driven by two significant factors [30]. The first is consumer awareness of environmental issues and preference for a healthy lifestyle. According to Mei et al. [31], natural preservatives have become increasingly popular for several reasons. They are natural and free from harmful chemicals, which minimizes the chances of adverse health problems. Technological advancement is another driver for market growth whereby the manufacturers competitively research active constituents to meet customer expectations [32]. Natural preservatives can be used as antimicrobial [33], antioxidant [2], and even anti-browning agents [9]. Furthermore, naturally derived additives can be used across a wide range of products, such as fruit, vegetable, meat, seafood, and others to preserve the natural characteristics of food and increase the shelf-life of food products for storage, which is economically important to avoid spoilage during transportation [3]. In Malaysia, fresh-cut fruit and vegetable products have been developed and sold in the market since long ago [34]. This is due to the increase in demand for fresh-cut fruit and vegetables in the market, which corresponds to the consumer’s preference towards ready-to-eat, good quality, and safe products [35]. Moreover, consumers perceive that fresh-cut fruit and vegetables are healthy and convenient, making them willing to pay a premium price [35]. The processing and handling of these fruits and vegetables have been structured to maintain the freshness of fruit and vegetable products [34]. The increased demand for convenience products has led to an increasing need for foods with a longer shelf life. Thus, it is predicted that Malaysia’s market demand for natural food preservatives will grow rapidly and will keep escalating in near future.

**Figure 3:**
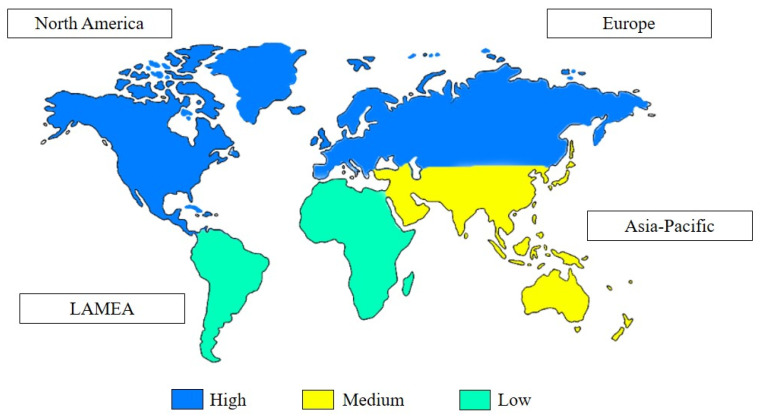
Forecast of natural preservatives market potential in North America, Europe, Asia-Pacific, and LAMEA regions from 2018–2026. Data gathered from Mordor Intelligence [37].

Based on Figure 3, the global natural preservatives market can be divided into North America, Europe, Asia-Pacific, and LAMEA (Latin America, Middle East & Africa). With the increasing demand for fresh-cut foods and rising vigilance towards health, the people of North America and Europe are utilizing more natural preservatives than other regions. This shows that North America and Europe emerge as dominant regions in the natural food preservatives market, a trend that is expected to continue in the future [36,37]. The increased availability of fresh-cut foods is also expected to aid market expansion in the Asia Pacific markets [36]. The trend towards natural preservatives has promoted the use of natural bioactive compounds produced from natural extracts, which is projected to positively impact the growth of natural preservatives markets.

### 1.3. Synthetic Versus Natural Sources

The fresh-cut produce market is growing rapidly each year due to the trend of modern lifestyles. With the increased attention of people to food safety, demand for fresh, healthy, inexpensive and additive-free food products has largely stimulated the expansion of the fresh-cut market [38]. In recent years, the food processing industry has progressively shifted to natural food preservatives for fresh produce because of the hazards of synthetic food preservatives.

The application of synthetic food preservatives must account for their side effects and potential hazards for human health. One of the possible harmful effects of synthetic food preservatives could act as a trigger for asthmatic reactions in sensitive individuals [10]. Sulfiting agents and their derivatives are primarily used as preservatives (to prevent the fruit and vegetables from discoloring) and as antioxidants in food industry [1]. Sulfiting agents, including sulfur dioxide, sodium metabisulfite and sodium sulfite are being used as chemical preservatives in various products. Despite their common utilization, sulfiting agents are considered as toxic molecules that are able to react with a variety of cellular components (proteins, lipids, DNA, etc) [39]. As noted previously, adverse effects of sulfiting agents on multiple organs of mammals, including the reproductive system [40,41] and the pulmonary system [42], have been reported. For some people who are hypersensitive to sulfiting agents, they may experience symptoms that range from mild to life-threatening reactions. Some of the symptoms include diarrhea, difficulty breathing, headache, vomiting, nausea, abdominal pain and cramps, which can be observed in sulfite-sensitive users [42,43]. These negative health effects have led to major concerns in the public. According to case studies, sulfite based-compounds have been documented to alter the oxidant and antioxidant balance in rat erythrocyte [44] and serum [43]. The alteration of the oxidant-antioxidant system makes it extremely poisonous to antioxidant defense system in adults due to a redox imbalance which leads to the accumulation of oxidative stress. An accumulation of oxidative stress in the body may further lead to cell and tissue damage and consequently results in the progression of many diseases [45].

The addition of antioxidant agents has also been widely practiced in food manufacturing to inhibit oxidation and enzymatic browning of fruit and vegetable. The use of antioxidants based-compounds in the human diet is one of the primary strategies [2]. Antioxidants are substances that can protect proteins, lipids and other biomolecules from oxidation, thereby preventing the formation of off-flavors in fruit and vegetable and thus increasing their shelf-life [45]. Several synthetic antioxidant chemicals such as butylated hydroxytoluene (BHT), butylated hydroxyanisole (BHA) and propyl gallate (PG) are added intentionally to fruit and vegetable [3]. Some of the chronic toxicity studies have found that BHT and BHA are able to damage the liver cell and have carcinogenic properties [9]. Due to the suspected action of these synthetic compounds, restrictions in the use of synthetic antioxidants have been enforced, given their health risks and toxicity. As a result, consumers have changed their preference and opted for natural food preservatives. The global market value for the natural food preservatives market was USD 796.1 million in 2018, and is projected to reach approximately USD 1068.1 million in 2026 [46]. The ever-expanding market for natural food preservatives and continual search for novel natural ingredients has led to various compounds or natural extracts including plants, herbs, fruits and animal by-products being studied as natural preservatives for fruit and vegetables [2,12].

Currently, the research on natural extracts with the potential of anti-browning and antioxidant properties to preserve fruit and vegetables from quality deterioration has increased worldwide and gained attention from all researchers all over the world. To satisfy the consumers, searching for additives of natural origin with additional health benefits has become an emerging interest. The use of natural substances from natural extracts with anti-browning and antioxidant properties would be of indubitable importance for a more widespread acceptance of food products by consumers [2]. The natural extracts can be obtained from fruits, vegetables, herbal, and animal by-products [1,2,3]. All these natural compounds can be applied as preservatives in fruits and vegetable products to ensure safety, protect their quality, and prolong their shelf-life. Besides, these natural compounds also contain high levels of bioactive substances that are consequently beneficial for health when the fruit and vegetable products are consumed [9]. Hence, using the natural extracts as food preservatives is a remarkable strategy to cater for the consumer demands and at the same time promote the innovation of creating value-added fruit and vegetable products.

## 2. Browning Control Mechanisms

**Figure 4:**
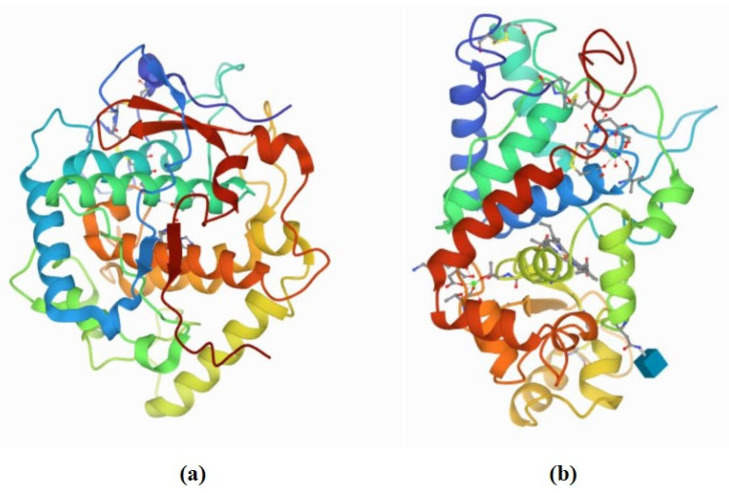
(**a**) Structure of grape (*Vitis vinifera*) PPO enzyme (Protein Data Bank entry: 2P3X); (**b**) structure of peanut (*Arachis hypogaea*) POD enzyme (Protein Data Bank entry: 1SCH).

**Figure 5:**
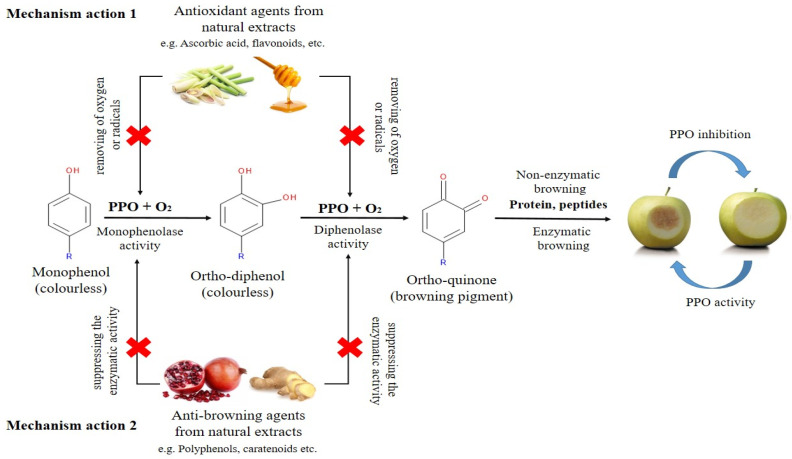
The schematic of enzymatic browning process and inhibition mechanisms of natural extracts. The figure was modified from Moon et al. [3].

Enzymatic browning is a ubiquitous phenomenon in fruits and vegetables caused by the oxidation of phenolic compound by PPO enzyme (Figure 4a). Generally, the PPO enzyme is classified into two groups, which are EC1.14.28.1 (tyrosinase, cresolase, and monophenol monooxygenase) and EC1.10.3.1 (*o*-diphenol oxygen oxidoreductase, diphenol oxidase, and catechol oxidase) [3]. These PPO enzymes are dicopper-containing enzymes, which contain two copper atoms at their active site and each bound with three histidines residues [47]. The two copper atoms in the PPO enzyme interact with the substrate and molecular oxygen resulting in the enzymatic browning process. The substrates that take part in enzymatic browning are located in plastids, while the PPO enzymes are found in the cytoplasm. When the plant tissue is injured or damaged, it w causes the plastids to rupture, resulting the PPO enzymes that localized in the cytoplasm to enter the plastids and interact with the substrates. Inside the plastid, the PPO enzymes will undergo two activities such as monophenolase and diphenolase activity (Figure 5). It involves the hydroxylation of monophenol (monophenolase activity) and the oxidation of ortho-diphenol to ortho-quinone (diphenolase activity). The ortho-quinone will then converted into complex brown polymers (melanin is formed) via the enzymatic and non-enzymatic reactions. These ortho-quinones are electrophiles, which can be easily attacked by proteins, peptides, amino acids, water, and other polyphenols, leading to Michael-type additions [3,48]. Enzymatic browning can only occur when all the essential reaction components are present. For this strategy, the inhibitory mechanism that aims to remove one or more of them presents a promising strategy. Therefore, the possible mechanism actions are: (i) removing of oxygen or radicals, or (ii) suppressing the enzymatic activity. Apart from that, POD (EC1.11.1.7) is another type of thermostable enzyme that responsible for oxidative browning reactions (Figure 4b) [6]. In the presence of hydrogen peroxide (H_2_O_2_), this oxidative POD enzyme will act by conducting single-electron oxidation on a wide variety of compounds, leading to enzymatic browning reactions. The POD reduces the H_2_O_2_ to water while oxidizing a variety of substrates [49]. For this reason, the catalytic reactions of the oxidative enzymes (PPO and POD) are important in controlling browning. Both enzymes possess some common substrates, and their common diphenolic substrates can lead to melanin formation. In addition, both enzymes have common inhibitors which can be used in preventing enzymatic browning [6,49]. For instance, polyphenol compounds are able to compete with the enzymes (PPO or POD) and interact at the active site of enzymes. Certain polyphenols compounds could also interact with proteins via hydrophobic interaction or hydrogen bonding [47]. These actions can eliminate the PPO or POD activity. Moreover, polyphenols possess hydroxyl groups that may be involved in electron donation to intermediate quinone, leading to a termination of the oxidation process. Besides, some enzymes derived from phenolic compound have been found to chelate the metal ions, especially Cu^2+^ at the binding and catalytic site of the enzyme. Consequently, enzyme activity becomes alleviated when these phenolic derivatives form hydrogen bonds at the active site of enzymes (PPO or POD) [47,50]. Therefore, investigating the ability of bioactive compounds from natural extracts to inhibit the PPO and POD activities in order to reduce the browning process is highly recommended.

## 3. Compounds Exhibiting Anti-Browning Properties

### 3.1. Polyphenols

Polyphenols are considered the largest group of chemical substances in fruits, vegetables, and herbs. They present in various form and serve as great anti-browning and antioxidant agents due to their multiple hydroxyl groups and phenol ring structures [3]. Polyphenols can be divided into three mains classes such as phenolic acids (gallic acid), flavonoids (flavone), and other phenolics (tannins). Phenolic-based compounds and their derivatives obtained from plant extract depict strong anti-browning and antioxidant properties and therefore present potential as natural inhibitors [2,9]. The natural inhibitors sourced from plants (phenolic rich extract) are free from harmful side effects and offer performance comparable to synthetic agents [31]. The benefits provided by plant extracts as natural preservatives, among others, such as low toxicity, low cost, biodegradability, renewability, etc. [12]. Many studies have reported on the utility of plant extracts from different plant parts (leaves, flowers, fruits, and seeds) as anti-browning and antioxidant additives for foods [12,13]. Various studies have also reported a strong positive correlation between the total phenolic content and antioxidant activity of plant extracts, which could prevent brown pigment formation in fruit and vegetable [51]. The antioxidant activity of phenolic compounds could be due to their redox properties, which allow the phenolic substances to act as reducing agents, singlet oxygen quenchers, and hydrogen donators [52]. Malaysia has a diverse range of plant species which could possess both potent anti-browning properties and antioxidant benefits for foods. The exploitation of natural plant extracts for the industrial production of natural preservatives is a fast-growing sector. These natural preservatives are expected to become very competitive in the market due to their higher biological value, and their potency as inhibitors of brown pigment formation. Importantly, these natural substances are not associated with cytotoxicity effects in human cells when compared to synthetic products [31]. Their effectiveness as natural anti-browning agents will be discussed in the following section.

### 3.2. Carotenoids

Carotenoids are plant pigments naturally present in many fruits and vegetables. Depending on the presence of oxygen in the carotenoid structure, they can be classified into two main carotenoids groups, namely oxygenated xanthophylls (zeaxanthin and lutein) and unoxygenated carotenes (lycopene, α-carotene, and β-carotene) [53]. Tomato and its by-products (skin) have been noted as an excellent source of carotenoids that may contribute to anti-browning activities [54,55]. Tomato varieties with a predominance of carotenoid contents have exhibited lower PPO activity [56]. For instance, lycopene and its derivatives act as an antioxidant agent that reconstructs the polyphenols oxidized by the PPO activity, resulting in reduced colour changes in tomatoes [55,56]. Martínez-Hernández et al. [55] prepared the lycopene microspheres obtained from tomato skin for reducing the apple browning. Thus, the application of carotenoids as an anti-browning agent in fresh-cut products is promising. Besides their anti-browning properties, carotenoids have been used as natural antioxidants for food product development in order to extend their shelf-life [54]. Carotenoids also play significant roles in the photosynthesis process by absorbing the light and protecting the organism from excessive light exposure, resulting in oxidative stress formation [57]. Hence, the application of carotenoids as an anti-browning agent in fresh-cut food products is highly recommended thanks to their ability to scavenge the free radicals associated with the development of browning.

### 3.3. Terpenoids

Terpenoids, also known as terpenes or isoprenoids, are secondary metabolites derived from natural sources. Terpenoids are ubiquitous, and can be found in nearly all living organisms. They are essential for plant growth and development and contribute the flavor, scents and colour [58]. Studies have reported that terpenoids found in citronella exhibit anti-browning activities [59], while Nakatsu et al. [60] reported that the terpenoids in the plant’s essential oil showed strong inhibitory effects on PPO activity in mushroom. The results obtained will be beneficial and can provide fundamental understanding for researchers in carrying out an extensive study in the future, and at the same time can encourage them to diversify the research by investigating the isolation and determination of the enzyme-inhibition activity of terpenoids extracted from different natural sources.

### 3.4. Organic Acids

Organic acids are a type of organic compound that possess weak acid properties which can be found naturally in plant and animal sources. Most of the organic acids are known as carboxyl acids, exhibiting anti-browning properties attributed to their metal-chelating activities or pH lowering effects [2]. They are great enzyme inhibitors as they able to deactivate the PPO enzyme and POD enzyme by lowing the pH in medium. A recent study highlighted that organic acid such as ascorbic acid, citric acid, and malic acid found in unripe grape were responsible for lowering the enzyme activity to prevent the browning development [61]. These organics are safe, with no restriction on their ingestion. Therefore, future studies are encouraged to search for the organic acids derived from natural sources in order to replace the chemical anti-browning agents that are available in the market.

### 3.5. Bioactive Peptides

Bioactive peptides are protein fragments that possess potential health benefits. Typically, it exists as a peptide residue with 2–20 amino acids and is inactive within the parent protein but can become active when it is released through fermentation, enzymatic or chemical digestion. Bioactive peptides are known to have various properties such as anticancer, antimicrobial, antihypertension, antidiabetic and antioxidative. Recently, bioactive peptides studies on preventing enzymatic browning have become a new trend. This is due to the fact that the antioxidants in bioactive peptides are capable of scavenging free radicals, donating electrons and chelating metals. One of the major factors that play a vital role in regulating their antioxidant activity is the amino acid composition. Previous studies have shown that bioactive peptides composed of hydrophobic amino acids (Methionine, Tryptophan, Phenylalanine and Proline) are crucial for the interaction with lipids, to facilitate superior radical scavenging activity, and one or more residues of Cysteine, Tyrosine and Histidine in their sequences [62,63,64,65]. Because of these, many food scientists have found that the protein hydrolysate (peptides) can be used to replace the sulfite agents for PPO inhibition [66]. From this perspective, bioactive peptides that improve food products’ shelf-life would be of utmost importance. Several recent studies showed that bioactive peptides from natural sources are able to suppress PPO activity, such as egg white [67], cod fish protein [68] and buffalo whey protein [69]. These bioactive peptides generated from natural sources serve as potential anti-browning agents due to their excellent antioxidant functions.

## 4. Extracts Derived from Fruit Sources

A great diversity of fruit species commonly available in market has caught the attention of food researchers due to their potential ability in controlling the enzymatic browning reaction. Therefore, natural extracts derived from fruit as well as agro-fruit by-products and wastes possess several bioactive compounds that act as anti-browning agents to alleviate the process of browning formation (Table 1).

Grape belongs to the Vitaceae family and is one of the new horticultural crops in Malaysia. These grapes are in the shape of oval or round fruit, with some being seedless. They possess a good taste with a sweet and sour flavour. Grape extracts are rich in catechin, and their total polyphenol contents depend on the variety of grape, degree of ripeness, soil type, climate, and environmental conditions [70]. Tinello et al. [61] determined the anti-browning and antioxidant properties of unripe grape (*Vitis vinifera*) juice. The authors noted that unripe grape juice exhibited strong anti-browning and antioxidant effects by significantly lowering the PPO activity, thus delaying the colour change of fresh-cut apple slices. The greatest potential anti-browning and antioxidant properties of grape juice was attributed to the presence of polyphenol compounds (catechin, chlorogenic acid, epicatechin, procyanidin B1 and B2) and organic acids (ascorbic acid, citric acid, malic acid), which were identified by high-performance liquid chromatography (HPLC). In a similar study also conducted by Tinello and Lante [71], they found that the unripe grape juice manifested not only the anti-browning but also antioxidant and whitening properties. They revealed that the unripe grape juice exhibited the greatest potential with tyrosinase inhibitory activity from 67.8% to 68.2%. At the same time, the unripe grape juice also showed the highest antioxidant capacity in DPPH (1 mg/mL) and FRAP (2 mg/mL) inhibitory activities. These properties were found to be beneficial, attributed to its high total phenolic contents (751.3–1961 µmol/L) and total organic acids contents (278.2–320.5 mmol/L).

Quince is the name given to the fruit of the *Cydonia oblonga* tree. A recent study investigated the use of quince seed extract to prolong the shelf-life of mandarin fruit (Table 1). Kozlu and Elmaci [72] reported that the fruit softening of mandarin fruit treated with quince seed extract was significantly delayed, weight loss was reduced, the discoloration of fruits when stored at 4 °C for 10 days was delayed. They also noted that the mandarin fruit treated with quince seed extract exhibited a satisfactory sensory evaluation in terms of appearance, texture, and taste. The quince seed extract showed a higher total phenolic content (4.05 mg/g) and antioxidant activity (40.48%) than the control (3.53 mg/g, 31.86%), without specifying the potential bioactive compounds that were responsible for these activities.

Cameron Highlands is the center of strawberry production in Malaysia. There are around 600 berry fruits in the world, of which the strawberry is the most popular berry fruit and is reported to contain bioactive properties of great interest for food industries [73]. The anti-browning activity of strawberries is associated with their high phenolic content, which includes phenolic acids, flavonoids, and phenolic compounds [2]. Dias and co-workers investigated the PPO and POD inhibition activities of methanolic extracts derived from strawberry leaves and branches [2]. The authors noted that both strawberry leaves and branches showed a significant effect in lowering the PPO and POD activity. There are 37 phenolic compounds from strawberry leaves and 33 phenolic compounds from strawberry branches which were tentatively identified. These bioactive compounds contain the hydroxyl group in the structure of phenolic compounds, whereby they interact by forming a hydrogen bond at the active site of the enzymes and thus reduce the enzyme (PPO and POD) activity. Apart from that, strawberry leaf and branch extract with free radical-scavenging activity, electron or hydrogen donating activity might contribute to anti-browning activity. For example, the phenolic compounds found in leaves and branches were gallic acid, procyanidin B type, catechin, epicatechin, and flavonols. These phenolic compounds have been proved to be effective PPO and POD inhibitors [47].

Coconut are Malaysia’s fourth largest industrial crop after oil palm, rubber and rice, with most plantations found in East Malaysia. The utilization of natural anti-browning agents for fresh-cut products has been recently considered. In a study conducted by Supapvanich et al. [74], apple wedges were immersed into coconut water, which significantly inhibited the PPO activity by at least 20% when compared to the control sample (without treated with coconut water). The authors also noted that the apple wedges dipped into the coconut water for 1 min exhibited a satisfactory color, appearance and decreased the yellowness and brownness values of samples during the cold storage (4 °C for 9 days). The total phenols concentration and antioxidant activity in fresh-cut apple wedges was increased by coconut water immersion. From this perspective, it is speculated that the total phenol contents and antioxidant activity of coconut water could play an important role in inhibiting the enzymatic reaction. Another study also investigated the efficiency of coconut water in preventing the browning incidence of fresh-cut apple as the fruit model [75]. The fresh-cut apple were immersed into different concentration of coconut water (0, 50 and 100%) for 2 min and then stored at 4 °C for 7 days. The whiteness of the fresh-cut apple was retained and the browning index was inhibited by immersion in 50% coconut water. With this evidence obtained, the coconut water could be a promising candidate as an anti-browning agent for fresh-cut fruit products.

Berries are well-known for their nutritional values since they possess a diverse variety of bioactive compounds such as polyphenols, carotenoids, vitamins and minerals for human well-being [76]. Investigations of Asian berries have identified strong anti-browning activity in the Moraceae family and the mulberry tree (*Morus alba*) is particularly attractive. The mulberry tree can be found in Asia, and it is widely cultivated in Japan, Korea and China for different uses. Various parts of this plant such as the twig, fruits, leaves, and root barks have been documented to have antioxidant, antimicrobial, anti-neurodegenerative, anti-inflammatory, and anticancer properties [76,77]. Recently, there has been an increased interest in the preparation of mulberry root barks and twig extracts as natural anti-browning agents in food systems (Table 1). There is a high amount of polyphenols compounds that can be found in the ethanol extract of mulberry root barks and twigs [78]. According to the characterization analysis on the components that are responsible for anti-browning properties via the chromatographic investigation, a high amount of polyphenol compounds and its derivatives, namely morin, rutin, resveratrol, maclurin, flavone were identified which showed the natural tyrosinase inhibitory and antioxidant effects [78,79]. Paudel et al. [9] isolated 2-Arylbenzofurans and its derivatives from mulberry root barks. An evaluation of these compounds on antioxidant and anti-tyrosinase activity has revealed their effectiveness against the DPPH free radical (IC_50_ ranging from 11.58 to 55.73 µM) and tyrosinase enzyme (IC_50_ value at 4.45 µM), showing its anti-browning ability. These benefits are attributed to the presence of the functional group prenyl, geranyl, or both, which enhance tyrosinase inhibitory and antioxidant effects.

Pomegranate, also known as *Punica granatum* L., is an edible fruit from the Punicaceae family. The fruit can be divided into three main parts, its peel (50% of the whole fruit), while the edible part consists of seeds (10%) and arils (40%) [80]. Pomegranate juice obtained from its fruit is the main element used in industry activity and production, other than direct consumption, their fresh arils and seed have also recently has gained industrial relevance. Due to a high demand for pomegranate juice, a large amount of waste products such as pomegranate peel has been discarded in public landfills, thus representing environmental concerns for the pomegranate processing industry. Therefore, extensive research is needed to change the traditional mindset that considers pomegranate peel as waste and leads to its disposal in landfills, to one that sees it as a superior bioresource reusable in the bio-economy, due to their high bioactive components and their diverse usage in different fields [81]. Recently, food scientists have proved that pomegranate peel possesses considerable amount of phenolic components and it has been related to antioxidant activity [82]. Therefore, the recovery and valorization of pomegranate peel with high antioxidant activity could be useful in controlling browning reactions. Turrini et al. [83] reported that the aqueous extract of pomegranate peels displayed DPPH free radical inhibition activity with IC_50_ values of 307 to 42 mg/g, and the tyrosinase inhibition activity was approximately 60%. The authors attributed the antioxidant and anti-browning properties to the high total phenolic content, which ranged from 148 to 237 mg/g. It is also speculated that the hydroxyl groups of the phenolic compounds in pomegranate peel extract could play a vital role in tyrosinase inhibition [47]. From this point of view, there is a positive correlation between phenolic contents and antioxidant activity. The high antioxidant capacity of pomegranate peel has shed light on its application as not only a natural anti-browning agent but as a health supplement rich in antioxidants.

Tomato *Solanum lycopersicum* (Family: Solanaceae), is a fruit of an herbaceous plant and represents one of the foods consumed worldwide. Since ancient years, tomatoes have been well-known for their rich antioxidant compounds such as phenolic components, carotenoids, and polyphenols, gifting to this fruit the recognition of functional food [84]. In addition, tomato skins generated during tomato processing, act as an excellent source of lycopene (carotenoid group) of approximately 14-fold higher than the pulp portion [3]. Tomato skins, due to their high lycopene content, have the potential to be incorporated as an antioxidant agent in anti-browning dipping treatments. A study successfully extract the lycopene content (7.23 g/kg) from the tomato skin and investigated their efficiency on preventing the browning formation of fresh-cut apples [55]. The authors noted that the fresh-cut apples that were immersed into the lycopene solutions (2 g/L) provided a satisfactory color and physicochemical properties, while maintaining a good microbial quality as well. They also highlighted that the immersion of a lycopene solution effectively controlled the browning reaction, showing the lowest browning index value after being stored for 9 days at 5 °C. Hence, the application of lycopene as an anti-browning agents in fruits is of high interest.

Mango (*Mangifera indica*) is a well-known tropical fruit which is important in terms of production and customer acceptance [85]. Natural components recovered from mango wastes or by-products can be used as an alternative strategy for controlling enzymatic browning. Mango peels are also considered as industrial waste during the mango processing process. The mango peels comprise around 7–24% of the whole fruit weight [86]. Therefore, the application of these valuable mango peels will definitely reduce waste management and environmental concerns. Moreover, mango peels have been reported to contain highly valuable compounds such as phenolic compounds, flavonoids and dietary fibers that is suitable to use as functional ingredients [87]. Phytochemical analysis on the mango peels clarified the presence of several bioactive compounds such as flavonoids (i.e., quercetin, anthocyanins, mangiferin), phenolic acids (i.e., gallic acid), and carotenoids, among other compounds with antioxidant properties [85]. Water extract from mango peel was able to retard the browning formation in potato puree [88]. Thus, it is evident that mango peel extracts have the potential to be utilized in natural anti-browning agents. The components isolated from the mango peel extracts, including mangiferin, gallic acid and protocatechuic, demonstrated the potent inhibition of PPO with an IC_50_ of 0.3 mg/mL. The anti-browning effects and functional properties of mango peels are a result of the high presence of these polyphenols derivatives [86]. Therefore, mango peels extract could be used as a natural anti-browning agent in the potato-processing industry.

Pineapple (*Ananas comosus*) is a famous fruit widely cultivated in many tropical and subtropical countries. They can be consumed either fresh or processed (i.e., canned beverages, fresh juice, jam, and jelly) [89]. Many previous studies have reported on the application of pineapple juice in inhibiting the enzymatic browning and maintaining the fresh-like quality of fresh-cut fruit [90]. The author noted that the pineapple juice effectively reduced the surface browning of fresh-cut apple, showing a lower browning value (browning index less than 1) than the untreated apple. When the fresh-cut apple was treated with 50% pineapple juice, enzymatic browning was inhibited for 6 days at 4 °C, without changing the physicochemical properties and maintaining the overall quality of fresh-cut apple. It is proven that the benefit of anti-browning properties in pineapple juice is attributed to the phenolic content, which has been reported to impart the browning inhibition property [91]. However, the phenolic compounds that are responsible for anti-browning activities have yet to be identified. Thus there is a need to further characterize the bioactive compounds in pineapple juice and identify other novel candidates that potentially inhibit the PPO or POD enzyme activity.

**Table 1:** Summary of most common natural sources from fruits, vegetables, plants/herbs, and others in market and their anti-browning properties reported within the last ten years.

Natural Sources	Bioactive Compounds/Extracts	Biological Activity	Mechanism of Action	Experimental Results	References
**Fruits sources**					
Unripe grape	Polyphenols (Caffeic acid, catechin, chlorogenic acid, gallic acid, epicatechin, epigallocatechin gallate), organic acids (citric acid, fumaric acid, malic acid, oxalic acid, succinic acid, tartaric acid)	Anti-tyrosinase, antioxidant	-Tyrosinase inhibition -DPPH and FRAP inhibition	The tyrosinase inhibitory activity varied between 67.8% and 68.2%. The grape juice showed the 1 mg/mL DPPH inhibition and 2 mg/mL FRAP inhibition. The anti-tyrosinase and antioxidant properties of unripe grape juices were attributed to the synergistic effects of polyphenols.	Tinello and Lante [71]
Unripe grape (*Vitis vinifera*)	Polyphenol (catechin chlorogenic acid, epicatechin, procyanidin B1 and procyanidin B2), organic acids (Ascorbic acid, citric acid, malic acid)	Antioxidant, anti-browning	-DPPH, ABTS and FRAP inhibition -Inhibit PPO enzymes	The unripe grape juice significantly inhibits the DPPH (3.62 mg/g), ABTS (6.43 mg/g) and FRAP (7.64 mg/g) activity. High antioxidant capacity induced high PPO inhibition activity (30%).	Tinello et al. [61]
Quince (*Cydonia oblonga*) seed	Quince seed extract	Antioxidant	-DPPH inhibition	Quince seed extract significantly reduced the rate of softening, maintained the color values, and reduced the weight loss (3.95%) of tested samples stored at 4 °C for 10 days. Quince seed extract exhibited a strong inhibition of DPPH activity (40.48%).	Kozlu and Elmaci [72]
Strawberry leaves and branches	Phenolic compounds (gallic acid, procyanidin B type, catechin, epicatechin, and flavonols)	Antioxidant, anti-browning	-ABTS inhibition -Inhibit PPO and POD enzymes	Strawberry leaves extract showed the inhibiting activity in ABTS (IC_50_: 0.65 mg/mL), PPO (IC_50_: 53.92 mg/mL) and POD (IC_50_: 0.77 mg/mL). Strawberry branches extract showed the inhibiting activity in ABTS (IC_50_: 0.75 mg/mL), PPO (IC_50_: 5.97 mg/mL) and POD (IC_50_: 2.25 mg/mL).	Dias et al. [2]
Coconut liquid endosperm	Coconut water	Anti-browning	-Inhibit PPO enzymes	Inhibition of PPO at least 20% during the cold storage (4 °C for 9 days).	Supapvanich et al. [74]
Coconut liquid endosperm	Coconut water	Anti-browning	-Inhibit PPO enzymes	The result revealed that visual appearance of the fresh-cut apple was maintained by 50% of coconut water immersion, which lowered the browning index, browning score, and maintained the whiteness index during the cold storage (4 °C) for 7 days.	Supapvanich et al. [75]
Mulberry (*Morus alba* Linn) root bark	2-arylbenzofuran	Antioxidant, anti-tyrosinase	-DPPH inhibition -Tyrosinase inhibition	The 2-arylbenzofuran derived from *M. alba* root bark manifested the good inhibitory of DPPH activity (IC_50_ ranging from 11.58 to 55.73 µM). The 2-arylbenzofuran exhibited good tyrosinase inhibition with IC_50_ value of 4.45 µM.	Paudel et al. [9]
Mulberry (*M. alba*) twigs	Polyphenol (flavone morin, rutin, resveratrol, maclurin)	Anti-tyrosinase	-Tyrosinase inhibition	The flavone compounds in *M. alba* twigs were found to inhibit tyrosinase activity with IC_50_ values between 0.07–8.0 μM.	Zhang et al. [79]
Pomegranate (*Punica granatum* L.) peel	Phenolic compound (Gallic acid)	Antioxidant, anti-tyrosinase	-DPPH inhibition -Tyrosinase inhibition	The IC_50_ for DPPH inhibitory activity varied between 307 to 42 mg/g. The tyrosinase inhibitory activity was approximately 60%.	Turrini et al. [83]
Tomato (*Solanum lycopersicum* L.) skin	Carotenoid (Lycopene)	Antioxidant, antimicrobial	-FRAP inhibition -Microbial growth inhibition	Lycopene of tomato skin showed FRAP inhibition activity with 824.0 mg/kg to 860.9 mg/kg. Lycopene treatment reduced the sample browning and inhibited the microbial growth after 9 days stored at 5 °C.	Martínez-Hernández et al. [55]
Mango (*Mangifera indica*) peel	Phenolic compound (Mangiferin, protocatechuic and gallic acid)	Anti-browning	-Inhibit PPO enzymes	The phenolic compounds of mango peels showed inhibitory effect on PPO with an IC_50_ of 0.3 mg/mL.	Jirasuteeruk and Theerakulkait [88]
Pineapple (*Ananas comosus*)	Phenolic compound	Anti-browning	N.M	High phenolic contents and reduced the browning development with low browning index (less than 1).	Supapvanich et al. [90]
**Vegetables sources**					
Ginger (*Zingiber officinale*)	Ginger extract	Anti-browning	-Inhibit PPO and POD enzymes	The ginger extract showed the 60.90% inhibitory activity of PPO and 48.10% inhibitory activity of POD.	Weerawardana et al. [92]
Potato (*Solanum tuberosum*) peel	Phenolic compound (caffeic acid and chlorogenic acid)	Antioxidant	-ABTS and FRAP inhibition	The potato peel extract showed ABTS and FRAP inhibitory effect with the total antioxidant capacity values of 0.21 μmol/mL and 0.28 μmol/mL, respectively. Reduced browning and slowed down the fruits softening can be observed during the storage for 3 days at 4 °C.	Venturi et al. [93]
Onion (*Allium cepa*)	Onion extract	Anti-browning	-Inhibit PPO enzymes	The onion extract manifested the good inhibition percentage on PPO activity (15.89–33.11%).	Lim and Wong [7]
Onion (*Allium cepa*)	Onion extract	Antioxidant	-ABTS inhibition	The onion extract displayed a good antiradical activity in ABTS (62.10%) after 30 days of storage.	Bustos et al. [94]
Onion (*Allium cepa*)	Polyphenols and flavonoids	Antioxidant, anti-browning	-DPPH and ABTS inhibition	The onion extracts exhibited higher DPPH inhibition activity (0.16 mmol/g) and ABTS inhibition activity (1 mmol/g). The onion extracts not only inhibited the browning development but also improved the nutritional quality of apple juice.	Lee et al. [95]
Chili pepper (*Capsicum* sp.)	Ascorbic acid	Anti-browning	-Inhibit PPO enzymes	The PPO activity inhibition by extracts of chili pepper was 70%.	Mercimek et al. [96]
Chili pepper (*Capsicum* sp.)	Chili pepper extract	Anti-browning	-Inhibit PPO enzymes	The chili pepper extracts showed the great potential of inhibition (45.97%) on PPO enzyme.	Lim et al. [97]
**Plants and Herbs sources**					
Mangrove plant leaves (*Bruguiera gymnorhiza*)	Polyphenols (Tannins)	Anti-tyrosinase, anti-browning, antioxidant	-Tyrosinase inhibition -DPPH, ABTS and FRAP inhibition -Inhibit PPO and POD enzymes	The tannins extracted from the *B. gymnorhiza* leaves exhibited strong anti-tyrosinase activity (IC_50_: 123.90 µg/mL). The IC_50_ value for antioxidant in DPPH, ABTS and FRAP were 88.81 µg/mL, 105.03 µg/mL, 1052.27 mg/g, respectively. Effective inhibited PPO and POD activity in fresh-cut lotus root stored at 4 °C for 17 days.	Liu et al. [98]
Mangrove plant leaves (*Bruguiera gymnorhiza*)	Flavonoids and phenolic compounds	Anti-browning	-Inhibit PPO enzymes	*B. gymnorhiza* leaves exhibited the greatest inhibition on sweet potato PPO (at least 50%).	Lim et al. [13]
Oregano Herb (*Origanum vulgare*) aerial parts	*O. vulgare* extract	Anti-browning	-Inhibit PPO enzymes	Prevention of enzymatic browning by PPO inhibition (64.50%).	Tanhaş et al. [99]
Citronella (*Cymbopogon nardus*) hydrosols	Terpenoids	Anti-browning	-Inhibit PPO and POD enzymes	Effectively suppressed the browning development by lowering the PPO and POD activities.	Xiao et al. [59]
Cinnamon	Cinnamon essential oil	Anti-browning	-Inhibit PPO enzymes	Decreased browning by inhibiting the PPO activity (80–90%).	Xu et al. [100]
Purslane (*Portulaca oleracea* L.)	Polyphenols and alkaloids	Anti-browning	-Inhibit PPO and POD enzymes	The 0.05% (w/w) purslane extract inhibited the PPO and POD activity in entire 8 days storage at 4 °C.	Liu et al. [68]
Purslane (*Portulaca oleracea* L.)	Purslane extract	Anti-browning	-Inhibit PPO and POD enzymes	Coupling the ultrasound treatment with purslane extract (0.02%) greatly promote the anti-browning effects on the fresh-cut potato across entire 8 days storage at 4 °C.	Zhu et al. [101]
Green tea leaves	Green tea extract	Anti-browning	-Inhibit PPO enzymes	Green tea extract (3 mg/mL) inhibit the PPO activity at least 86%.	Klimczak and Gliszczyńska-Świgło [102]
Green tea leaves (*Camellia sinensis*)	Flavonoids (catechins)	Anti-browning	-Inhibit PPO enzymes	Green tea extract showed high PPO inhibitory activity at least 80%.	Chang and Kim [103]
Aloe (*Aloe vera*)	*A. vera* extract	Anti-browning	-Inhibit PPO and POD enzymes	The fresh-cut fruits treated with *A. vera* extract showed the lowest browning score (1.94%), reduced the PPO and POD activity across entire 6 days storage at 4 °C.	Supapvanich et al. [104]
Stevia plant (*Stevia rebaudiana*) leaves	Stevia leaves extract	Anti-browning	-Inhibit PPO and POD enzymes	Reducing the PPO and POD activity with the incubation time when the Stevia leaves extract were added.	Criado et al. [105]
**Others**					
Manuka Honey	Honey extract	Anti-browning	-Inhibit PPO enzymes	The inhibitory effect of honey extract on PPO activity varied from a range of 41.39% to 48.0%.	Lim et al. [97]
Egg white	Peptides	Antioxidant, Anti-tyrosinase	-DPPH, and ABTS inhibition -Tyrosinase inhibition	The bioactive peptide derived from egg white exhibit the good antioxidant activity in DPPH (20%), ABTS (0.3 mg/mL) and tyrosinase inhibition (IC_50_: 2.90 mg/mL).	Thaha et al. [67]
Cod fish skin	Peptides	Anti-browning	-Inhibit PPO and POD enzymes	The cod fish derived bioactive peptides significantly inhibit the PPO and POD activity during the entire 8 days storage at 4 °C.	Liu et al. [106]
Buffalo whey	Peptides	Antioxidant, Anti-browning	-ABTS inhibition -Inhibit PPO enzymes	The buffalo whey peptides exhibited significant increase in antioxidant activity and PPO-inhibitory activity (50% inhibition).	da Silva et al. [69]
Blue mussel (*Mytilus edulis*)	Organic compound (Hypotaurine and sulfinic acids)	Anti-browning	-Inhibit PPO enzymes	The hypotaurine and sulfinic acids compound found in blue mussel extract exhibited great inhibition of PPO activity (ranging from 89% to 100%).	Schulbach et al. [107]

## 5. Extracts Derived from Vegetables Sources

Many researchers have focused on searching for potential natural anti-browning agents from natural products that prevent enzymatic browning and improve the nutritional value of food. Of these natural products, vegetables are well known for their excellent sources of secondary metabolites with known anti-browning and antioxidant activities, including polyphenols, flavonoids, phenolic acids, carotenoids, proteins, and terpenes which have been gaining interest as a nutritional strategy to prevent browning [1]. In line with this, Table 1 summarizes studies related to controlling the browning process of using the natural extract derived from distinct vegetable sources.

*Zingiber officinale*, popularly known as ginger, is a very common and widely used vegetable in cuisine. Apart from cooking, ginger has always had some other usage, such as in candy, ayurveda, digestive juices and certain relevant remedies to prevent nausea and vomiting [108]. Ginger belongs to the Zingiberaceae family. It is a source of the polyphenols components such as 6-gingerol and its derivatives contain high antioxidant properties [109]. Therefore, it has been explored for its PPO and POD inhibitory potential. Weerawardana et al. [92] investigated the PPO and POD inhibition activities of the aqueous extracts of ginger. The ginger extract showed a significantly higher percentage of inhibitory activity, of 60.90% on PPO activity, and 48.10% on POD activity. However, the bioactive compounds responsible for the anti-browning effects are unknown. Therefore, research should look to elucidate the contribution of different bioactive compounds found in the aqueous ginger extracts to their PPO and POD inhibition activities.

One of the most important food crops is potato (*Solanum tuberosum*), which can be consumed from fresh to processed formulations (i.e., potatoes chip, mashed potatoes, etc.) thus fueling the increase in waste generation by the food manufacturing process [110]. Potato crop is one of the most highly consumed vegetables, however, potato peel is the major waste from the potato processing industry. For this reason, an interesting source of natural bioactive compounds might be found in the waste derived from the potato processing industry. Recently, Venturi et al. [93] proposed natural enzyme inhibitors by using the potato peel extracts as antioxidant additives for fresh-cut fruits. The authors noted that the potato peel extracts showed an ABTS and FRAP inhibitory effect with the total antioxidant capacity values of 0.21 μmol/mL and 0.28 μmol/mL, respectively. The firmness of fresh-cut apples treated with the potato peel extracts remained unchanged and prevented the softening of flesh tissue during cold storage for 3 days at 4 °C This resulted in slowing the rate of browning formation on fresh-cut apple. The advantageous effect exerted by potato peel extracts could correspond to their phenolic content. Phenols and its derivatives have shown different behaviors in controlling browning reactions. The phenolic components found in potato peel extracts include caffeic acid and chlorogenic acid, which have been proven to suppress PPO activity [93,111]. The result revealed not only a benefit for potato-peel waste management by could also help to increase the shelf-life of other fresh-cuts fruits and vegetables.

Onion (*Allium cepa*) is an essential part of the diet and commonly used in cuisine worldwide. It occupies a significant place in human nutrition. In this regard, onion has been recognized as a great source of healthy flavonoids (flavonoes and quercetins) and sulfur-containing compounds (allyl trisulfide, diallyl sulfide, and allyl-cysteine) [112]. Lee et al. [95] reported that apple juices treated with onion extracts exhibited better functionally, which markedly improved the antioxidant capacity, total polyphenols and flavonoid content. The addition of onion extracts also significantly retards browning and enhances the radical scavenging activity of apple juice. Similarly, Bustos et al. [94] noted that onion extracts improved the antioxidant capacity of the avocado pulp by almost 60%. The authors speculated that the antioxidant capability of onion extracts could be attributed to the sulfur-containing compounds and their derivatives. Lim et al. [97] studied the PPO inhibitory effects of the extracts derived from onions. It was reported that the aqueous extract of onions exhibited a good inhibition effect on PPO activity (15.89–33.11%). The onion extracts contained the sulfhydryl group, which enable allows for their interaction with copper at the PPO active site, thus hindering the PPO catalytic activity [112]. From this aspect, natural inhibitors derived from onion extracts can be utilized to replace unsafe chemicals such as synthetic sulphite-containing gents, which could present a threat to human health.

Chili pepper (*Capsicum* sp.) is an economically important commercial crop and it is grown in almost all regions across Malaysia. Mercimek et al. [96] found that potato PPO inhibition activity by chili pepper extracts was 70%. The authors speculated that the high PPO inhibitory activity of chili pepper extracts could be attributed to the high ascorbic acid content found in the chili pepper (approximately 1.44 mg/g of chili pepper). In another study, Lim et al. [97] examined the inhibitory effects of chili pepper extracts on the PPO activity of sweet potato. The authors also noted that chili peppers extract exerted the strongest inhibition on sweet potato, with values of 45.97%. The authors speculated that PPO-inhibition activity and anti-browning effects were due to their phenolic compounds, flavonoid and carotene, which act as natural antioxidants [7]. It was also hypothesized that the PPO-inhibition activity of the chili pepper extracts was due to their ascorbic acid via the reduction of ortho-quinone back to ortho-diphenol. Hence, these speculations still need further investigation. These findings suggest thar chili pepper extracts might be a novel alternative to synthetic PPO inhibitors in the food processing industrial.

## 6. Extracts Derived from Plants or Herbs Sources

Several natural inhibitors have been established from plants or herbs, showing their merits in preventing browning formations. The application of herb extracts and their derived biochemical substances has likely been a recent trend for controlling the enzymatic browning of fruits and vegetables due to their PPO-inhibition potential and anti-browning capacity. As summarized in Table 1, there is a wealth of evidence suggesting that the extracts derived from plants or herbs are potentially effective anti-browning agents.

Mangrove plants play an important role in coastlines protection. *Bruguiera gymnorhiza* (Family: Rhizophoraceae) is one of the species in the mangrove family that can be found in Malaysia. This mangrove plant has been recorded in traditional medicine, where it is commonly used in diabetes and hypertension management [113]. Recent studies reported that *B. gymnorhiza* not only has pharmacologic potential but also has shown inhibitory effects towards PPO and POD of fruits and vegetables (Table 1). Liu et al. [98] noted that the predominant tannins isolated from *B. gymnorhiza* leaves are promising anti-browning agents for fresh-cut food products. The authors evaluated the antioxidant activities of tannins compounds from *B. gymnorhiza* leaves, whereby the IC_50_ value for DPPH, ABTS, and FRAP was 88.81 µg/mL, 105.03 µg/mL, 1052.27 mg/g, respectively. The authors also demonstrated that tannins from *B. gymnorhiza* leaves had excellent effects on suppressing the tyrosinase, PPO, and POD, protecting the fresh-cut lotus root from browning. Similarly, Lim et al. [13] also claimed that *B. gymnorhiza* leaves are potential sources of natural PPO inhibitors. The authors showed that *B. gymnorhiza* leaves manifested the greatest inhibition on sweet potato PPO (at least 50%), which was attributed to flavonoids and phenolic compounds such as quercetin, coumarin, and gallic acids. These bioactive compounds have been documented as powerful antioxidant agent, which could chelate the copper at the PPO active site and prevent the catalytic of enzyme activity. Together, these benefits result from the evidence that *B. gymnorhiza* leaves could be useful in controlling the enzymatic browning of fruits and vegetables, and they can be integrated into the industrial process.

Oregano herb (*Origanum vulgare*) is an aromatic plant of the Lamiaceae family, which has been well developed for controlling foodborne pathogenic-bacteria growth [114]. Their use as natural agents for food preservation provides a novel way to improve the quality and shelf-life of fresh-cut fruits and vegetables. Tanhaş et al. [99] investigated the alternative usage of natural inhibitors derived from the aerial part of the oregano herb in inhibiting the PPO activity. The authors noted that the extracts from the aerial parts of the oregano herb exhibited the strongest PPO inhibition, of 64.5%. The PPO inhibition activity of the oregano herb extract was attributed to their polyphenols and their derivatives, so they presumably have antioxidant properties against enzymatic browning [115]. However, future studies are recommended to examine the contribution of the different polyphenols derivatives compounds in the oregano herb that contribute to the PPO- and POD-inhibition activities. In such a view, the utilization of natural preservatives such as an aqueous extract from oregano herb could be a great alternative approach to minimize the chance of fresh-cut foods deterioration and preserve their qualities.

The important citronella grass species, such as *Cymbopogon nardus*, popularly known as serai Wangi (Malay), have been used in the traditional medicine and herbs market for centuries [116]. They have been widely cultivated in most tropical Asian countries including Malaysia. Being the most popular plant in Malaysia, depending on the specific uses, this citronella grass is well known for its antioxidants and consists of some interesting biochemical substances that can prevent the browning reaction. Liu et al. [68] reported the presence of one major component, terpenoids, with antioxidant properties in citronella grass hydrosol (by-products of essential oil from steam distillation). The authors showed that the citronella hydrosol effectively retarded the browning of fresh-cut taros. The PPO and POD enzymes were inhibited, which could be due to the terpenoids constituents which exerted around 35.54% in citronella hydrosol. These terpenoids compounds acted as a competitive inhibitor of PPO, protecting the plant against enzymatic browning, inhibiting the action of the enzyme and thus maintaining fresh-cut color and texture [117]. With these beneficial effects, the citronella hydrosol could be useful in reducing the enzyme activity of fresh-cut foods.

Cinnamon is a favorite household spice and is used worldwide. It is not only used for cooking but also in modern and traditional remedies. Cinnamon, similarly to other plants, contains a variety of bioactive compounds that exhibit antimicrobial properties [118]. There is some evidence that cinnamon essential oil, with antimicrobial properties, can be applied in celery and melons [119,120]. They are an excellent source of essential oil and can provide antimicrobial properties in the food system, while also contributing to antioxidant and anti-browning effects against enzymatic browning. Xu et al. [100] investigated the effect of cinnamon essential oil on the PPO activity in apple juice. The results revealed that the PPO activity was inhibited by about 80–90% by the cinnamon essential oil, resulting in fewer ortho-quinones generated by enzymatic browning. However, although the study reported that the cinnamon essential oil has PPO inhibition activity, it is still not clear what potent components in the essential oil are responsible for the anti-browning actions. Therefore, future studies are suggested to elucidate the contribution to PPO inhibition activity of the essential oils.

Purslane (*Portulaca oleracea* L.) is a widespread plant that is used not only as an edible plant, but also as a traditional medicine to reduce the risk of human diseases such as severe inflammation, fever, headaches, gastrointestinal and bladder ulcers [121]. As a natural antioxidant agent, the properties of purslane extract are inseparable from the alkaloids and polyphenols in their composition. In research conducted by Liu et al. [68], the efficient concentration of purslane extract was found to be 0.05% (*w*/*w*), and achieved its strongest inhibition activity on PPO and POD during 4 °C storage for 8 days. Extract derived from purslane has effectively inhibited the fresh-cut potato browning, attributed to their contents of thirty polyphenols and fifty-six alkaloids, which were identified in purslane extract. Similarly, Zhu et al. [101] also found that a low concentration of purslane extract (0.02%, *w*/*w*) could effectively prevent the browning with the aid of ultrasonic treatment. The authors showed that the integration of ultrasound treatment with the purslane extract could significantly suppress the PPO and POD activities, and also provide the satisfactory appearance of fresh-cut potato slices. This may be due to the fact that the ultrasound enhanced the extraction process by inducing the cavitation to break down the plant cell (purslane extract) and promote the bioactive compounds released into the solvent, resulting in a low dose of purslane extract that could provide an anti-browning effect. These factors indicate that the combination of sonication and natural extract could be a promising strategy for controlling the enzymatic browning in fresh-cut industry.

Green tea (*Camellia sinensis*) is one of the most common drinks that can be found in the world. Green tea is a rich source of polyphenols and natural antioxidants used as alternatives to chemical antioxidants [122]. These natural antioxidants, which prevent the oxidation of organic molecules, are very critical for food preservation. Chang and Kim [103] noted that the ethanolic extract of green tea exhibited PPO inhibition activity (80%). The authors reported that the PPO inhibition activity correlated with the flavonoid compounds, such as catechin. It has been speculated that catechin presumably acted a competitive inhibitor for PPO because of its structure similarity, which was able to stop the PPO reaction. In another study, Klimczak and Gliszczyńska-Świgło [102] also clearly reported that the effectiveness of green tea to suppress the PPO reaction in apple juice was 3 mg/mL. After 48 h, the green tea extract at 3 mg/mL inhibited the PPO reaction by 86%. The authors speculated that the PPO inhibition might be attributed to the polyphenols, such as catechin, which have been documented to act as strong competitive PPO inhibitors. From this aspect, the enzymatic browning of fruits and vegetables can be effectively inhibited by the green tea extract in order to extend the shelf-life of the products.

Aloe (*Aloe vera*) from the Liliaceae family has been well developed and used in the cosmetic field, due to its whitening effect [123]. The utilization of *A. vera* as a natural anti-browning agent is an alternative way to retain the quality and inhibit the browning in minimally processed foods. An extract obtained from *A. vera* gel was studied as an anti-browning agent in fresh-cut apple [104]. The authors found that the fresh-cut apples supplemented with 75% of *A. vera* gel manifested reduced browning and increased the total phenolic content, antioxidant capacities, and maintained the sensorial quality of the fresh-cut apple. However, the compounds from *A. vera* gel that act against the brown pigment formation is still ambiguous and further investigation in needed to elucidate the mechanism by which the bioactive compounds found in *A. vera* gel regulate their anti-browning activity.

Stevia plant (*Stevia rebaudiana*) is usually used for sweeteners in product formulation. Interestingly, stevia leaves provide an excellent source of antioxidant components that could influence the key oxidative enzymes (PPO and POD) activity [124]. Noteworthy is the study that used stevia plant leaves extract as anti-browning agents in orange, mango, and papaya [105]. The authors noted that PPO and POD activity was greatly affected by the addition of the stevia leaves extracts, showing that the stevia leaves extracts have an ability to inhibit the enzyme activity. The authors speculated that the PPO and POD activity was inhibited by the two main phenolic and flavonoid compounds present in stevia leaves. In this sense, stevia-leaf extract could play a significant role in reducing oxidation and could act as an anti-browning agent to prevent food deterioration.

## 7. Extracts Derived from Other Sources

In recent decades, natural anti-browning agents derived from animal by-products have attracted interest due to their PPO inhibition capability and their anti-browning capacity [125]. It has been hypothesized that enzyme inhibitors derived from honey, fish skin, egg white, whey, and mussels offer an alternative strategy to control the browning in fruits and vegetables. Lim et al. [97] noted that honey could be the best natural inhibitor for sweet potato PPO, as it exhibited the highest PPO inhibition (41.49% to 48%) as compared with standard ascorbic acid. The authors speculated that honey possesses flavonoids derivatives that have been proven to have antioxidant properties, which allowed for chelation with copper atoms in the PPO active site [7]. Another possible reason identified by the author is the presence of small peptide (less than 600 kDa) in honey, which promotes its inhibition by reducing the ortho-quinones or by acting as copper chelators of the active site of PPO [66]. Furthermore, numerous studies on bioactive peptides for promoting human health and enhancing the shelf-life of food products have become available. The bioactive peptides derived from fish skin [106], egg white [67] and whey [69] have been well studied as inhibitors of the PPO enzyme, showing potential for applicability. The potent anti-browning properties of bioactive peptides could be attributed to their good antioxidant function [126]. Liu et al. [106] found that 0.1% (*w*/*w*) bioactive peptide from codfish skin was significantly inhibited enzymatic browning via the suppression of PPO and POD activity. The authors speculated that peptides could suppress enzyme reactions by forming the enzyme-inhibitor complexes. The peptides have a similar structure to the enzyme-substrate, which, as a chelating agent, is able to react with the ortho-quinones and produce a colorless adduct to prevent browning [126]. Moreover, the bioactive peptide derived from egg white revealed superior antioxidant activity in DPPH (20%), ABTS (0.3 mg/mL) and tyrosinase inhibition (IC_50_: 2.90 mg/mL) [67]. The authors also noted that peptides from egg white have the ability to prevent browning formation by immobilizing the substrate. Meanwhile, da Silva et al. [69] also showed that the browning inhibition effect of buffalo whey peptides effectively reduced browning formation in fresh-cut apples. The authors noted that the peptides obtained from buffalo whey, which are structurally similar to PPO substrate, form a strong interaction with the copper at PPO active site, which would cause competitive inhibition [66]. These actions stabilize the ortho-quinones, blocking subsequent reactions that form the brown pigment. Other sources from marines such as mollusks also have been highlighted as potential inhibitors for browning inhibition. Schulbach et al. [107] noted that the blue mussel (*Mytilus edulis*) extract contains compounds such as hypotaurine and other sulfinic acid derivatives (benzene and methane sulfinic acids), which act as antioxidants and inhibitors of the enzymatic browning of foods. The authors showed that the hypotaurine and sulfinic acids compound found in blue mussel extract exhibited a great inhibition of PPO activity (ranging from 89% to 100%). From this perspective, the inhibitor found in the animal by-products showed good capability in inhibiting the enzymatic browning. Since the oxidative enzymes (PPO and POD) are responsible for losses to fresh-cut foods, natural inhibitors derived from animal by-products that prevent browning might be valuable. Recovered and valorized food wastes or by-products that suppress browning and an understanding of their action mode could be beneficial to industrial processing [106,107]. Inhibitors from natural sources ingested by consumers are more acceptable as natural ingredients.

## 8. Potential Application of Genome-Editing in Preventing Browning Activity and Improving Postharvest Quality

Enzymatic browning is an unwanted but unavoidable mechanism that presents a significant hurdle for both producers and industry. It may influence the food quality and appearance during harvest and post-harvest activity such as shipping, storage and distribution [3]. The industry has applied several approaches to control this undesired effect via physical and/or chemical methods. These methods can easily regulate the browning process by inhibiting the PPO enzymatic activity [3]. However, the use of these methods to control enzymatic browning and at the same time maintain the quality of fresh-cut products is challenging, due to the growth in consumer demand for foods that are safer, healthier and chemical-free, and also better for the environment.

The manipulation of plant genomes in a precise manner is one of the innovations in gene-editing technologies to improve crop quality, especially in post-harvest management. Over the decades, extensive investigation has been carried out, leading to new genome-editing tools to produce exact and desired plant modifications. Oligonucleotide Directed Mutagenesis (ONM) is one of the earliest gene-editing tools that has been developed in in plant science research over the decades, and Engineered Nucleus (ENs) has been primarily used over the past 15 years [127]. For instance, endonucleases/ meganucleases (EMNs), zinc-finger nucleases (ZFNs), TAL effector nucleases (TALENs) and clustered regularly interspaced short palindromic repeats (CRISPR) [128,129,130] are examples of gene-editing tools that have been successfully applied to produce valuable traits in many crop species. Besides, genetic modulation by an exploitation of the function of small or micro RNA (miRNAs) that target the PPO genes have also been extensively studied to uncover a potential candidate for genetic engineering to prevent or reduce browning activity in fruits and vegetables. Among these engineered nucleases methods, TALENs and CRISPR System are the most widely used techniques in plant genome editing [127].

### 8.1. Current Application of CRISPR/Cas Technology in Preventing Browning

Clustered regularly interspaced short palindromic repeats and CRISPR-associated proteins or CRISPR/Cas is a recently advanced gene-editing tool that has been used for plant genome-editing due to its potency, versatility, adequacy and simplicity. It consists of CRISPR repeat-spacer arrays and Cas protein, a naturally occurring mechanism mediated by RNA in a prokaryotic system that protects the organism against phages or other invasive genetic material by breaking the nucleic acid of the invader [131,132]. The first application of CRISPR/Cas9-based genome editing in the plant was recorded back in 2013 [133,134,135], and since then, the CRISPR/Cas9 system has become a flexible approach for genome-editing research. In the most current approach, the CRISPR/Cas9 system in plant genome-editing has become the method of choice because the simple modification method is applicable for most common crop plants. Moreover, the application is not merely predicted to be used in plant breeding programs and in post-harvest management to enhance storage and shelf life, but it is also able to maintain the quality of fruits and vegetables.

Many reports have discussed the application of important agronomic traits in plant genomes using the CRISPR/Cas9 system, and one of the recent applications of the CRISPR/Cas9 system in preventing enzymatic browning was reported by González et al. [128]. They adapted the CRISPR/Cas9 system to create a mutation in the *StPPO2* gene in potato, with the objective to lower the PPO activity and subsequently reduce enzymatic browning. They employed a Ribonucleoprotein complex (RNPs) to transfect the potato protoplasts by introducing two sgRNAs to direct Cas9 nuclease [136]. Verification using High-Resolution Fragment Analysis (HRFA) has shown that the selected lines successfully induced mutations only in the *StPPO2*, without altering coding sequences of other *StPPOs* gene family and consequently their roles in other cell functions and systems. Besides, an analysis of relative enzymatic browning and relative PPO activity also demonstrated a significant reduction of enzymatic browning activity and tuber PPO activity of up to 75% and 69%, respectively. This finding proved that the technology applied (CRISPR/Cas9 system) was able to increase the efficiency of inducing mutation at the specific target gene, whilst minimizing the insertion of foreign DNA into the target sites.

In a different study, Maioli et al. [129] identified ten gene- encoding PPO proteins (*SmelPPO1*-*10*) from eggplant berry. Out of ten genes, they characterized three *PPO* target genes (*SmelPPO4, SmelPPO5* and *SmelPPO6*) that demonstrated high transcript levels in eggplant berry after cutting. They employed CRISPR/Cas9-based mutagenesis to knockout class B *PPO* genes, which are responsible for wounding stress and the defence response, to obtain plants with low flesh-browning genotypes. This was performed by identifying a conserved region of target genes corresponding to the tyrosine domain and designing a gRNA targeting *SmelPPO4*, *SmelPPO5* and *SmelPPO6.* The construct was then transformed into the eggplant cotyledons and the mutations were screened in the generation plants. The selected lines carrying mutations in *SmelPPO4*-5-6 were tested for a phenotypic analysis of enzymatic browning and PPO activity in berries and were compared to wild type. Based on observations after 30 min of cutting and exposure to air, it was found that brown discoloration was more prominent in wild type and the average PPO activity was significantly decreased by up to 52% compared to wild type. This study showed that genetic editing via CRISPR/Cas9-mediated knockout of PPOs is a promising approach for developing eggplant varieties with low browning activity and, at the same time, could preserve their antioxidant, nutrition and phenolic content after the post-harvesting procedure.

Overall, the CRISPR/Cas9 system has become a rapidly growing technology in genome editing research. Extensive investigation in this field has established the application of the technology from a bacterial adaptive immune system to its advancement and employment as the most efficient and straightforward approach. The objective is to design a genome-editing tool that is not only applicable for prokaryotes and eukaryotes but also for plants, especially in preventing the browning activity to promote post-harvesting improvement. For decades, the system was proven to be applicable when adapted to the prokaryotic, mammalian and plant systems. Its contribution to fundamental studies and investigating the functional categories at molecular and protein levels has been recognized extensively. Importantly, CRISPR/Cas9 has served as a new technology that is used to disable genes coding for PPOs and represents the most promising strategy to avoid undesired browning in plant-derived products. It has successfully transformed the potentialities of research into reality by generating specific changes in target genes with a simple alteration at the DNA level.

### 8.2. Challenge in the Application of CRISPR/Cas9 System in Crop Plants

Although the CRISPR/Cas9 system has been acknowledged for its benefits in plant breeding and genetic modification to improve plants quality, there is still some major concerns in introducing this system to crop plants. This is because CRISPR/Cas9 modified plants may be considered as genetically modified organisms (GMOs), which are banned in some countries. However, two alternative strategies have been proposed to potentially overcome this problem, which involve introducing both Cas9-encoding mRNA and gRNAs or pre-assembled ribonucleoprotein complexes. Nevertheless, the efficiency of the transient mutation using this method is relatively low and not comparable to the conventional CRISPR/Cas9 technology. This is due to the fact that transient expression strategies via plasmid delivery have always had limited applicability as most plant species are not susceptible to these DNA-delivery methods [137] Thus, further justification and modification of the existing technology are needed to ensure that the CRISPR/Cas9 system is not labeled as GMO.

Another drawback of CRISPR/Cas9 system is the off-target effect of the CRISPR/Cas9 system itself. This happens because of the limited large-scale data available to assist the study on the off-target effect of CRISPR/Cas9 in a plant. Despite having some disadvantages as mentioned above, CRISPR/Cas9 is still the method of choice in plant breeding due to its promising technology, which can be applied to integrate various economically important traits in plants. Besides, the technology is proven to be effective in improving the genotype and phenotype of various crops, thereby leading to better yields that can meet the needs of the world population.

### 8.3. Other Application of Genome-Editing Tools in Preventing Browning Effect

Furthermore, microRNAs (miRNAs) are small noncoding RNAs generally 20 to 24 nt long, and play an essential role as a gene regulator in the transcription process during the cleavage and/or translation inhibition of the target mRNAs. In plants, miRNAs are involved in many critical biological processes and their function is conserved almost in all systems or life cycles including growth development, flowering, fruit ripening, postharvest effects such as browning and senescence, and stress signaling [138,139]. Recent findings found that plant miRNAs are also associated with the browning activity of postharvest fruits and vegetables. This reaction is regulated by the PPO, which is the primary enzyme responsible for the browning effect in fruits and vegetables. Previously, researchers identified eight miRNAs targeting PPO encoding genes, which might be responsible for the suppression of brown spots on potatoes [140] and osa-miR2923a, which inhibit PPO activity and consequently prevent browning reactions [141]. These reports showed that the combination of miRNAs and their target genes to form complexes played an important role in regulating browning activity.

**Figure 6:**
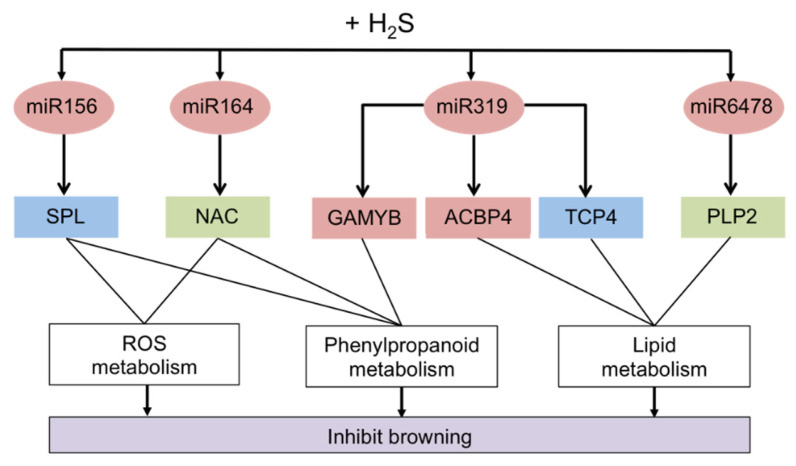
A proposed schematic model of miRNA-mediated browning inhibition fresh-cut apples induced by H_2_S. Red colour represents the DEmiRNAs or target genes that were down-regulated, blue colour represents the up-regulated target genes while green colour represents the target genes that either up-regulated or down-regulated.

Recently, Chen et al. [138] reported the use of miRNA in regulating the browning effects in fresh-cut apples. They uncovered that the inhibition of browning activity in fresh-cut apples is regulated by hydrogen sulfide, by employing 12 small RNA libraries of H_2_S-treated and control fresh-cut apples at two storage times together with one mixed degradome library. They identified the differentially expressed miRNAs (DEmiRNAs) that are responsible for the disruption of browning activity by H_2_S and discovered the possible roles of the DEmiRNAs and target genes that contributed to the inactivation of the browning process. They also performed transcriptome sequencing to investigate the expression dynamic of the target genes of DEmiRNAs (*SPL*, *NAC*, *GAMYB*, *ACBP4*, *TCP4* and *PLP2*). They hypothesized that the perturbation of ROS, phenylpropanoid, and lipid metabolism is associated with H_2_S treatment that suppressed the expression of miRNAs and their target genes, resulting in the disruption of the browning process in fresh-cut apples (Figure 6). These findings provide novel insights into the molecular basis and a deeper understanding of the underlying browning inhibition by H_2_S treatment [138].

**Figure 7:**
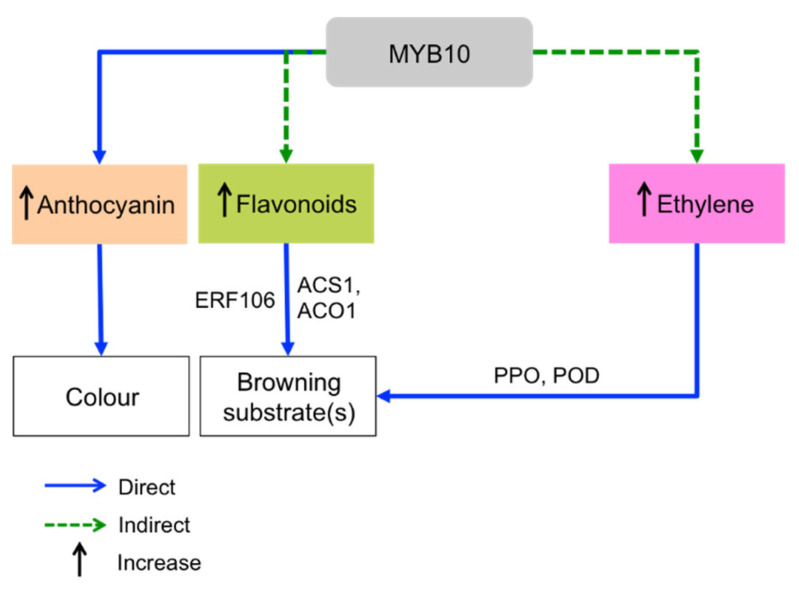
A proposed schematic model to elucidate the link between increased anthocyanin level and internal browning-flesh disease activation. The expression of MYB10 TF directly increases anthocyanin levels by activating the anthocyanin biosynthesis pathway, which indirectly induces flavonoid production by enhancing pathway flux. Furthermore, MYB10 is also associated with the early activation of ethylene production possibly via interaction with ERF106. Early activation of ethylene production leads to an increase in transcription and enzyme activity caused by PPO. These events consequently promote early ripening, production of POD enzyme and accumulation of additional substrate(s) which caused browning.

The elevation of the anthocyanin contents in fruits and vegetables is a breeding target for many crops. In some fruit, such as tomato, higher anthocyanin concentrations enhance storage and shelf life. In contrast, highly anthocyanic red-fleshed apples (*Malus* x *domestica*) have an increased incidence of internal browning flesh disorder (IBFD). Espley et al. [142] used the over-expression of anthocyanin-related transcription factor (TF) MYB10 (35S:MYB10), which resulted in the production of highly pigmented flesh in ‘Royal Gala’ apples, to investigate the mechanism of IBFD in WT ‘Royal Gala’. However, they found that the over-expression of MYB10 in the same genotype increased the incidence of IBFD. Besides, their analysis of some important genes associated with ethylene biosynthesis (aminocyclopropane-1-carboxylic acid synthase and oxidase; ACS and ACO) and PPO displayed a significant increase in the ethylene level and the mechanism for enhanced PPO-mediated browning. Besides, the expression level of the transcription factor of the ethylene response factor (ERF) class, ERF106, was also up-regulated in red flesh. An investigation on transcriptional activation, mediated by MYB10, showed that this transcription factor promotes the activation of apple ACS, ACO, and ERF106 genes expression. The ERFs responsible for the termination of the ethylene signaling cascade could results in either the activation or disruption of ethylene production and at the same time regulate the expression of ripening-related genes. In addition, they elucidated a possible link related to the elevation of anthocyanin-related transcription factors and internal browning flesh disorder. They also presented a proposed model that described the activation of MYB10 TF, which may up-regulate the anthocyanin biosynthetic pathway, thus increasing the anthocyanin concentration and indirectly promoting the production of flavonoid (Figure 7). The MYB10 TF was also hypothesized to interact with ERF106, leading to premature ethylene production and eventually early ripening [142]. In conclusion, this study provides evidence for a better understanding of a possible link between the regulation of ripening-related genes and for preventing browning activity which improves the fruits’ shelf life and storage management.

Li et al. [143] reported the manipulation of the plant genetics of Tomato in 2019. They studied the gene responsible for fruit ripening as it involves a series of interactions between a complex biological system of both endogenous and exogenous factors. This reaction resulted in a series of biochemistry and physiology changes, such as color deterioration, fruit softening, aroma volatiles evolution, nutrients composition and so on [143]. Many researchers have been worked on a different type of transcription factor and discovered the role of each transcription factor in regulating fruit ripening in the system, such as MADS-RIN [144], Nor [145,146], TAGL1 [147], and zinc finger transcription factor SlAFP2 [148]. In their study, Li and his group investigated the role of long non-coding RNA known as lncRNA1459, which is associated with a ripening-related RNA in tomato. Although there was previous work discussing and characterizing the function of lncRNA in general, the role of lncRNAs in fruit ripening remains ambiguous. Their recent investigation employed the CRISPR/Cas9 system to delete the lncRNA1459 gene and create CR-lncRNA1459 mutants. They managed to identify the subcellular of IncRNA1459 from successfully cloned full-length lncRNA1459. Based on their finding, two homozygous CR-lncRNA1459 mutant lines that work independently displayed a significant delay in ripening by disrupting ethylene production and preventing the accumulation of lycopene, which is comparable to the wild type (WT) fruits. Following this discovery, they performed an RNA- seq analysis to investigate the different expression genes (DEGs) and different expression lncRNAs (DELs) of CR- lncRNA1459 mutants and WT. The results showed that lncRNA1459 plays a role in tomato fruit ripening, and this finding provided a new direction to develop a better understanding of the lncRNA’s function in fleshy fruit ripening. These results may also provide some fundamental information for further investigation and for the manipulation of this ripening-related gene in anti-browning-related studies.

## 9. Method of Incorporations with Natural Extracts in Preventing Browning

There are various innovative approaches that can be used as eco-friendly systems for effectively reducing enzyme activities (PPO and POD), thereby preventing enzymatic browning and consequently enhancing organoleptic, nutritional and antioxidant properties of food products. The food industry is continually looking for methods to reduce food waste and spoilage. The most common techniques, such as dipping treatments and edible coatings have been implemented in combinations with the use of natural extracts to control the enzymatic browning process in fresh foods produce. Tinello and Lante [1] highlighted that natural extract can be used alone or in combination with other anti-browning agents in fresh foods produced by dipping treatments or via their incorporation in edible coating formulations. Therefore, these techniques offer the advantages that allow enzyme inhibitors to penetrate specific target enzymes (PPO and POD) located underneath the cell membranes or other organelles in order to increase the efficacy in the dark-pigment prevention of foods.

The dipping technique is one of the popular ways to control the food browning phenomena after the postharvest processing, as peeling or cutting processes are completed in the fresh-cut industry. This dipping treatment can either suppress the PPO and POD enzyme activities or their substrates. Hence, surface treatment by dipping fresh fruit and vegetables in natural extract solutions with anti-browning/antioxidant properties is widely practiced so as to improve the shelf-life and the safety of foods. Besides, another major concern in fresh-cut products is related to their contamination by foodborne pathogens and spoilage microorganism. Nevertheless, to control the microbial growth, it is common to apply dipping treatments with natural extract solutions. Scientists have proven natural extracts consist of natural antimicrobial agents that can therefore provide a natural alternative to food additives. These natural extracts contain a wide range of volatile compounds and some of them are important flavor quality factors. They are also classified as Generally Recognized As Safe (GRAS) compounds and permitted as food additives that are able to demonstrate the inhibition and inactivation of microbial growth. Several studies have mentioned the application of natural extract via the dipping treatment in foods such as poultry meat [149], seafood [150], fruit [151] and vegetable [7]. Natural dipping treatments may exert direct effects on microbial growth, such as an inhibition of metabolic respiration, the disruption of cell walls and a modulation of the microorganism activities.

Dipping application could be considered highly suitable due to the enzymatic browning process, which can be inhibited by the direct immersion of food pieces into a natural extract solution of anti-browning properties. However, coating is another technique that can be used to minimize undesirable changes in foods. The application of edible coatings has emerged as a technique in food processing and preservation to improve food safety and extend the shelf-life of the food. The incorporation of natural resources in edible coating formulations is considered a potentially safe option that is able to enhance the quality of fresh produce. The said coatings are thin layers of edible substances applied to the food produce surface, thereby providing an effective barrier and eventually inactivating the enzyme and microorganisms activities. Ncama et al. [152] also highlighted plant-based edible coatings as one of the eco-friendly approaches free of any detrimental side effects if consumed at a concentration effective for maintaining product quality. The application of plant-based edible coatings has been studied by a number of food technologists and interest in this research was intensified with the introduction of fresh-cut products in the market. The plant-based edible coating is usually based on extracts obtained from plants that are regarded healthy for human consumption such as herbs, fruits or spices with high antimicrobial and antioxidant properties. Its use has been found in its potential of conserving the quality of various food products including fruits [153], vegetables [154] and meat- and fish-derived products [155]. Here, it is worth mentioning that plant-based edible coatings are able to inhibit the enzyme (PPO and POD) activity and reduce browning along with preserving the quality of the treated commodity.

The extraction technologies used to obtain the bioactive compounds with anti-browning and antioxidant properties from natural sources are fairly important. Novel browning alleviation technologies for fresh-cut fruits and vegetables such as edible coating films, high-pressure high oxygen, and microwaves have been used to control the browning development [1]. However, these technologies require sophisticate instruments that are expensive, which become major drawbacks for implementation [38]. The other emerging innovations for extraction in modern technology is ultrasound-assisted extraction (UAE) that contributes some advantages such as convenience, ease to operate, time-saving, and high extraction efficiency with less energy [22]. Apart from that, UAE is considered a non-thermal and eco-friendly approach and its application in controlling the browning of fresh-cut products has been well developed [1,12]. Previously, synthetic anti-browning agents such as ascorbic acid, cysteine, and calcium ascorbate, which are associated with negative impacts on human health and are not environmentally friendly have often been used in the UAE method to prevent browning formation in foods [156]. Due to safety concerns, the integration of UAE with natural extracts, which extends the shelf-life of fresh-cut products, has been established recently. Zhu et al. [101] reported that the incorporation of purslane extract with UAE is an effective way to inhibit browning development by suppressing the activities of PPO and POD. Jirasuteeruk and Theerakulkait [88] found that the utilization of mango peel extracts along in the UAE treatment helps to retain the quality of fresh-cut potato and reduces the rate of browning formation. An important element in the use of UAE is that it can enhance the bioactive compound recovery from the natural sources via the cavitation phenomenon. The cavitation effect can be explained in four steps [157]. First, cavitation bubbles are produced near the plant substrate surface when ultrasound is applied. Second, the bubble bursts and releases the pressure and temperature of the micro-jet to the surface of the plant material. Third, the matrix surface is destroyed, and direct contact is established between the intracellular active ingredient and the external solvent. Finally, the active ingredient is released and delivered to the solvent. In this way, the ultrasound can enhance the mass transfer and increase the concentration of active ingredients extracted, thus preventing oxidation via the inhibition of the PPO enzyme. Therefore, this UAE can work with any liquid medium, and the solvents do not limit its advantages [12]. Future studies are encouraged to optimize the use of bioactive compounds in the UAE of antioxidant and anti-browning from natural extracts by means of the optimization process.

## 10. Patents

A patent is an intellectual property (IP) right for a scientist’s invention. Patents encourage research and development (R&D) activities, benefiting society by creating useful products and services that improve the quality of life [158]. R&D investment activities are also expected to obtain IP for innovative findings, as IP satisfies the criteria of global novelty and industrial application. Patent search and analysis from the Google Patents and Lens Organization (https://www.lens.org/) (accessed on 27 October 2021) have been performed using the keywords “natural” AND “anti-browning” AND “fruit” AND “plant”. A brief description of the patents and findings are presented in Table 2. Specifically, those inventions that focus on the utilization of natural sources that confer a delay in browning, lower the total microbial count, improve the general organoleptic properties, and decrease the amount of chemical contaminants on the exposed food surfaces without the use of synthetic preservatives are presented. Apart from that, countries such as the United States, South Korea, Australia and China also presented interesting patent technologies related to natural sources, all with great potential for use in the food system. They can be used to preserve food, beverages, and other food products because of the anti-browning and anti-oxidant properties of natural sources found in these countries. However, Malaysia is still a long way from translating these inventions into commercially available products. The numerous patents published in recent years can further promote research and innovative opportunities in Malaysia.

## 11. Conclusions and Future Research Prospects

At the turn of this century, natural sources from fruits, vegetables, plants/herbs, and animal by-products with anti-browning properties received increased attention among scientists for the discovery of effective and safe anti-browning agents against food browning. Consumers’ habits of food intake patterns are changing rapidly as well. Consumers all around the world are becoming more preoccupied with the food they eat. They prefer to eat food that is healthy, organic, nutritious, and environmentally friendly. Hence, the green concept is now steadily being propagated among consumers in conjunction with the sustainability and conservation of agricultural development. In line with the concept, the utilization of natural extracts as an alternative to synthetic food additives for fresh food products preservation has attracted more attention due to the fact that natural extracts are interesting prospects in the array of anti-browning agents. These natural food additives from those substances have the possibility of gaining trust from the consumers. It is true that natural extracts are being used to increase the functional properties of a vast variety of food products; however, the application of natural extracts as anti-browning agents on food products without negatively affecting the organoleptic characteristics is still a challenge for food technologists. This led to the introduction of the genome-editing strategy as a potential tool to be applied for reducing enzymatic browning and thus improving the postharvest quality of horticultural crops. Our review revealed that different natural sources that are commonly available in market have the potential to preserve their quality and delay the enzymatic browning activity. Meanwhile, our review revealed that the manipulation of plant genomes in a precise manner could also be utilized to reduce the browning effect and hence improve the crop quality. Nevertheless, safety is the primary concern for an enzyme inhibitor to be used in food processing and production either via physical, chemical or genetic approaches.

Continuous exploration related to improving enzyme inhibitors by all approaches is highly recommended. It would be valuable to investigate the mechanisms associated with the mode of action and the preservative effect of these natural inhibitors during the food preservation process. For example, the exploitation of biological processes via genetic manipulation or gene editing technology has been a potential approach to reduce the browning effect. However, it has become a significant challenge because it relies on the understanding of the exact mechanism of these pathways so that it can be beneficial for preventing browning activity. Because the technique is relatively cheap and easy, with minimal impact on the genome, the cost barrier is such that for the first time, scientists can feasibly engineer postharvest traits with the expectation that the new germplasm could be commercially viable. This means that despite the challenges that have been outlined, it should be noted that the exploitation of the plant genome to control the browning activity in fruits and vegetable is possible. Reports on such approaches in recent days have illustrated the feasibility of obtaining numbers of a homozygous variety in a single mutation that is heritable and stable without integrating unwanted foreign DNA. For instance, gene knock-out or silencing using CRISPR/Cas9 technology and the manipulated miRNAs expression of its regulatory genes by up-regulating or down-regulating a specific transcription factor involved in several metabolic and biosynthesis pathways (i.e., anthocyanin), have been proven to reduce enzymatic browning. The development of these technologies to control PPOs activity in fruits and vegetables has become one of the promising approaches for undesirable browning management. The application of either one of these gene-editing technologies, or in combination, is not only beneficial for maintaining the antioxidant and nutritional properties during the post-harvest procedure but also could possibly pass the restrictions assessing regulatory matters regarding genome-edited organisms. For example, González et al. [128] demonstrated that the developed technology successfully rescued enzymatic browning in tubers and could produce edited potato plants that do not fall under strict GMOs regulation and would consequently benefit farmers, industries and consumers. As discussed above, genetic manipulations have been reported to provide reliable phenotypes for reducing browning activity, and there are others that are very promising. There are still many ongoing studies being carried out to elucidate the outcome of using gene editing technology, such as prolonging shelf-life or improving the quality of major commercial crops.

Edible coatings on food products has become the new trend and has found increasing demand. More research aimed at determining the application of plant extracts as edible coatings are warranted. Plant-based edible coatings can be applied to protect active components, such as colorants, anti-browning components, flavors, etc. Coatings are able to increase the shelf-life and reduce the risk of food spoilage by microorganisms. However, research on fresh-cut fruit and vegetable is insufficient, and their industrial application is still under process and remains widely unknown, which leads to the need for further demonstration in future. The aspects that affect their industrial implementation should also be taken into consideration, such as costs, registration requirements, etc. Another consideration that can be made when applying the plant-based edible coating on food products’ surfaces is their ability and the period of adhesion to the products’ surface. Furthermore, the application of nanotechnology such as the nano- and micro-encapsulation of active constitutes can be a suitable technique to improve the effectiveness of natural extracts. The proteomics and transcriptase research should also include studies on the extensive spectrum of protein and gene expression profiles, which can help to develop a greater understanding of the natural extracts’ target in food preservation, thereby providing information on the affected physiological pathways and important receptor activity. Apart from that, partnership and collaboration across the food industry, academia and government regulatory agencies are key for the success of the application of natural anti-browning agents from plants in pilot scales, especially in the coupling of UAE technologies with natural extracts. Adopting proper statistical techniques to optimize their application methods to achieve a higher efficiency may help the industry sectors to overcome the hurdles currently faced that preclude a more widespread and effective use of natural food additives on a larger scale in food preservation. Thus, more research should also be performed towards the discovery of novel natural anti-browning agents, so as to expand the range of products available for the development of mixtures with advanced preservation properties for food applications.

## Figures and Tables

**Table 2:** Patents for recently explored studies on natural sources with anti-browning properties.

Patent Number	Approval Year	Country	Natural Sources	Patent Title	Summary of Inventions
CN111066878A	2020	China	Fruit and vegetable	Natural fruit and vegetable preservative and preparation process thereof	This patent related the natural fruit and vegetable extract as a potential fresh-keeping agent.
US6224926B1	2019	United States	Pineapple	Natural anti-browning and antioxidant compositions and methods for making the same	This patent describes active ingredients containing pineapple juice as a browning and oxidizing inhibitor.
AU2017260170A1	2017	Australia	Acerola cherry	Juice products and methods for reduced enzymatic browning	This patent is related to the anti-browning potentials of acerola cherry fruit to reduce the oxidation process produced by PPO.
US20160338368A1	2016	United States	Green tea	Fruit and vegetable preservative	This invention describes the preservation of fruits and vegetables using green tea extract.
US20140127369A1	2014	United States	*Chrysanthemum indicum*	Composition containing *Chrysanthemum indicum* L. extract for preventing discoloration	This patent is related to anti-browning agents containing the extracts of *Chrysanthemum indicum* for preventing browning of fruits and vegetables.
KR20120135778A	2011	South Korea	Citrus peel	Anti-browning agents containing the extracts of pericarp of *Citrus linn*	This invention relates to the utilization of citrus-peel extract for browning prevention.
KR101300603B1	2011	South Korea	*Chrysanthemum indicum*	Anti-browning agents containing the extracts of *Chrysanthemum indicum*	This invention relates to browning prevention by using the active ingredient extract from *Chrysanthemum indicum.*

## Data Availability

Not applicable.
